# Xeno interactions between MHC-I proteins and molecular chaperones enable ligand exchange on a broad repertoire of HLA allotypes

**DOI:** 10.1126/sciadv.ade7151

**Published:** 2023-02-24

**Authors:** Yi Sun, Georgia F. Papadaki, Christine A. Devlin, Julia N. Danon, Michael C. Young, Trenton J. Winters, George M. Burslem, Erik Procko, Nikolaos G. Sgourakis

**Affiliations:** ^1^ Department of Pathology and Laboratory Medicine, Children’s Hospital of Philadelphia, Philadelphia, PA 19104, USA.; ^2^ Department of Biochemistry and Biophysics, Perelman School of Medicine, University of Pennsylvania, 3501 Civic Center Blvd., Philadelphia, PA 19104, USA.; ^3^ Department of Biochemistry and Cancer Center at Illinois, University of Illinois, Urbana, IL 61820, USA.; ^4^ Department of Cancer Biology and Epigenetics Institute, Perelman School of Medicine, University of Pennsylvania, Philadelphia, PA 19104, USA.

## Abstract

Immunological chaperones tapasin and TAP binding protein, related (TAPBPR) play key roles in antigenic peptide optimization and quality control of nascent class I major histocompatibility complex (MHC-I) molecules. The polymorphic nature of MHC-I proteins leads to a range of allelic dependencies on chaperones for assembly and cell-surface expression, limiting chaperone-mediated peptide exchange to a restricted set of human leukocyte antigen (HLA) allotypes. Here, we demonstrate and characterize xeno interactions between a chicken TAPBPR ortholog and a complementary repertoire of HLA allotypes, relative to its human counterpart. We find that TAPBPR orthologs recognize empty MHC-I with broader allele specificity and facilitate peptide exchange by maintaining a reservoir of receptive molecules. Deep mutational scanning of human TAPBPR further identifies gain-of-function mutants, resembling the chicken sequence, which can enhance HLA-A*01:01 expression in situ and promote peptide exchange in vitro. These results highlight that polymorphic sites on MHC-I and chaperone surfaces can be engineered to manipulate their interactions, enabling chaperone-mediated peptide exchange on disease-relevant HLA alleles.

## Teaser

Finetuning interactions with chaperones can broaden the scope of MHC-I allotypes that are amenable to antigen repertoire editing.

## INTRODUCTION

Class I major histocompatibility complex (MHC-I) proteins display epitopic peptides on the cell surface, thereby providing a basis for immune surveillance by T cell receptors (TCRs), which can recognize aberrant peptides and mediate CD8^+^ cytotoxic responses against infected or malignant cells (*1*). MHC-I folding and peptide loading are subject to intricate cellular quality control. The peptide-loading complex (PLC), comprising the transporter associated with antigen processing (TAP), the molecular chaperones tapasin, ERp57, and calreticulin, assembles peptide–MHC-I (pMHC-I) molecules with high-affinity peptides in the endoplasmic reticulum (ER) (*2*–*5*). In addition, TAPBPR, a homolog of tapasin, which functions outside the PLC (*6*, *7*), plays a complementary role in pMHC-I optimization and quality control (*8*, *9*). Although both tapasin and TAPBPR function as peptide editors (*9*–*12*), TAPBPR also participates in the reglucosylation cycle of MHC-I molecules by promoting interactions with uridine 5′-diphosphate–glucose:glycoprotein glucosyltransferase 1 (*13*). These unique functions of tapasin and TAPBPR ultimately lead to an optimized repertoire of stable pMHC-I molecules for cell-surface trafficking and interactions with TCRs.

The classical HLA loci (human leukocyte antigen, the human MHC) encode the most variable proteins in the human genome with more than 35,000 alleles (*14*). Polymorphic residues in the HLA peptide–binding groove define a unique peptide repertoire displayed by each allotype (*15*), enabling species adaptability to emerging infections. HLA-A, HLA-B, and HLA-C allotypes show divergent dependencies on molecular chaperones for proper assembly and cell-surface expression, which has important biological ramifications. Tapasin independence of MHC-I alleles correlates with an increased breadth of the peptide repertoire (*16*) and can lead to enhanced control of HIV viral loads (*17*). Likewise, TAPBPR-knockout (KO) cell lines express MHC-I molecules presenting a broader spectrum of peptides, relative to wild-type (WT) cells (*18*). Amino acid variations in the α_2_ and α_3_ domains can affect interactions with tapasin and cell-surface expression levels (*19*, *20*). While polymorphic residues located on the floor of the MHC-I groove lead to a gradient of tapasin dependencies for HLA-B alleles (*21*), TAPBPR preferentially interacts with HLA-A over HLA-B and HLA-C alleles, where specific residues H114 and Y116 confer gain-of-function binding to TAPBPR when introduced to noninteracting HLA allotypes (*22*). Some of these effects can be explained by the interacting surfaces observed in the x-ray structures of chaperoned, peptide-deficient MHC-I complexes (*23*, *24*), including a conserved allosteric site underneath the α_2–1_ helix revealed by solution nuclear magnetic resonance (NMR) (*12*). Furthermore, highly polymorphic MHC-I residues distant to the chaperone binding sites can influence interactions with TAPBPR by modulating the dynamic sampling of an “open” conformation (*25*, *26*). Notwithstanding, chaperones can recognize a much broader allelic repertoire of partially folded MHC-I molecules, as shown by deep mutagenesis experiments (*25*, *27*). This adaptability of underlying interactions suggests that the corresponding conformational epitopes on folded MHC-I molecules are suboptimal for binding to chaperones. Notably, while, in human, the antigen-processing genes are removed from the HLA-I loci, close gene association in the chicken MHC has led to coevolution of antigen-processing and class I genes, causing mirrored polymorphisms in TAP and tapasin that segregate with specific MHC-I alleles (*28*–*30*). The orthologous TAPBPR gene was initially identified in mice and humans and later in fish and chickens, strongly suggesting a conserved function (*31*). Although structural modeling studies suggest a similar overall protein fold (*32*), functional differences among TAPBPR orthologs have not yet been characterized, with most studies concentrating on the human protein.

Here, we characterize and contrast the allelic specificity and molecular and functional features of HLA interactions with TAPBPR orthologs from *Homo sapiens* (human), *Gallus gallus* (chicken), and *Mus musculus* (mouse). We find xeno reactivity between chicken TAPBPR (chTAPBPR) and multiple HLA allotypes that are not competent for binding to human TAPBPR (hTAPBPR), such as HLA-A*01:01 and HLA-B*08:01, and demonstrate direct interactions with peptide-loaded or -deficient molecules covering all six classified HLA-A supertypes, B08 and B44 supertypes (*33*, *34*), as well as HLA-E, HLA-G, and MHC-I related (MR1). Deep mutational scanning of hTAPBPR expressed at the plasma membrane identifies gain-of-function mutants that mimic amino acids found in the chTAPBPR sequence and greatly enhance peptide exchange function on HLA-A*02:01 while also enabling exchange on HLA-A*01:01. Overall, our results underscore a strong correlation between the capacity of TAPBPR variants to bind empty molecules and maintain them in a peptide-receptive state, with catalytic peptide exchange function in vitro. Our findings highlight the plasticity of recognition surfaces on MHC-I and TAPBPR molecules, which can be used to fine-tune interactions. Insights from our work can be leveraged to enable peptide exchange technologies for a wider range of disease-associated HLA allotypes, including nonclassical MHC-I molecules.

## RESULTS

### Divergent TAPBPR orthologs show a range of stabilities and interactions with MHC-I molecules

**Fig. 1.**
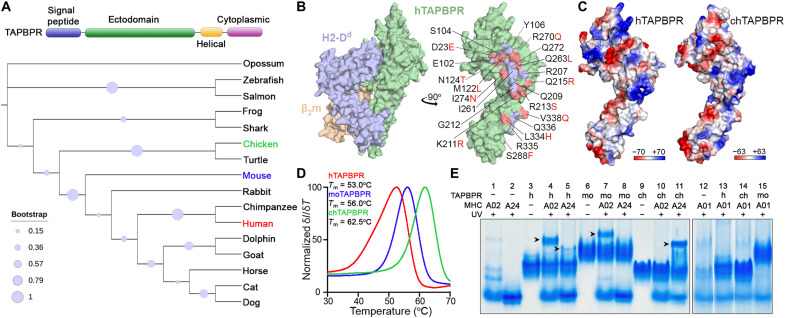
Divergent TAPBPR sequences show differences in stability and recognition of HLA allotypes. (**A**) Phylogenetic analysis of TAPBPR ectodomain sequences from selected species. Evolutionary analysis was conducted in MEGA7. The bootstrap values (500 bootstrap trials), depicted as spheres, indicate the percentage of trees in which the associated taxa clustered together. (**B**) Surface representation of hTAPBPR in contact with mouse H2-D^d^/hβ_2_m complex (PDB ID: 5WER) (left) (*24*). The predicted contact residues of hTAPBPR are highlighted in purple or red for the conserved or polymorphic residues between human and chicken orthologs, respectively (right). For the polymorphic sites, the respective chicken residues are shown in red. (**C**) Comparison of the electrostatic potential surfaces for hTAPBPR and chTAPBPR in the indicated ranges (down to −70 *k*_B_*T*/*e* in red and up to +70 *k*_B_*T*/*e* in blue, where *k*_B_ is the Boltzmann constant, *T* is the temperature, and *e* is the unit charge) as calculated using the Adaptive Poisson-Boltzmann Solver (APBS) solver in PyMOL (*74*). The structural model of chTAPBPR was generated using the BAKER-ROBETTA server (*72*). (**D**) *T*_m_ (in degrees Celsius) obtained from DSF for recombinant hTAPBPR, chTAPBPR, and moTAPBPR. The data shown represent replicate assays (*n* = 3). (**E**) Native gel shift analysis of TAPBPR orthologs and MHC-I complex formation. Each well is loaded with pMHC-I (A02, HLA-A*02:01 refolded with KILGFVFJV; A24, HLA-A*24:02 refolded with VYGJVRACL; and A01, HLA-A*01:01 refolded with STAPGJLEY), where J = 3-amino-3-(2-nitrophenyl)-propionic acid, TAPBPR, or 1:1 molar ratio mixture. Samples were UV-irradiated for 1 hour at 365 nm, when indicated. The formed MHC-I/TAPBPR complexes are indicated with arrows.

To identify evolutionarily divergent TAPBPR orthologs with unique features relevant to interactions with MHC-I molecules, we performed a phylogenetic analysis focusing on the ectodomains of 16 TAPBPR sequences from different species (Fig. 1A). hTAPBPR and chTAPBPR share 46% sequence identity with regions of relatively higher conservation. Mouse TAPBPR (moTAPBPR) is an intermediate between the two (77/50% sequence identity relative to human/chicken; fig. S1). The structure of hTAPBPR interacting with mouse H2-D^d^/human β_2_-microglobulin [hβ_2_m; Protein Data Bank (PDB) ID: 5WER] illustrates that 12 of 23 residues in contact with the MHC-I heavy chain (*24*) differ between hTAPBPR and chTAPBPR (Fig. 1B). Structure modeling of chTAPBPR based on an hTAPBPR/murine MHC-I cocrystal structure (*24*) followed by an electrostatic surface potential analysis reveals divergent features of MHC-I interaction footprints on TAPBPR (Fig. 1C). Specifically, contiguous surfaces formed by residues 16 to 20, 35 to 40, 139 to 145, and R270 define positively and negatively charged epitopes in the hTAPBPR and chTAPBPR sequences, respectively (Fig. 1, B and C). Together, the sequence and structure-based analyses highlight the potential for xeno interactions between TAPBPR orthologs and complementary sets of HLA-I.

We next compared the biochemical properties of different TAPBPR orthologs expressed as recombinant proteins and purified by size exclusion chromatography (SEC) (fig. S2). Differential scanning fluorimetry (DSF) analysis showed a substantial increase in the thermal stabilities of mouse and chTAPBPR, with melting temperatures (*T*_m_) of 56 and 62.5°C, compared to hTAPBPR (*T*_m_ = 53°C) (Fig. 1D). We further evaluated interactions of different TAPBPR orthologs with representative common HLA allotypes HLA-A*01:01, HLA-A*02:01, and HLA-A*24:02 using an electrophoretic mobility shift assay (EMSA) performed under native conditions. Here, we characterized interactions with peptide-deficient MHC-I molecules, as previous studies showed the formation of high-affinity protein complexes (*11*, *23*, *24*). While producing empty MHC-I molecules was challenging because of their instability (*35*), we refolded HLA molecules with photosensitive peptides, which could dissociate upon ultraviolet (UV) irradiation generating empty molecules (*36*, *37*). Different HLAs (lanes 1, 2, and 12) and TAPBPR orthologs (lanes 3, 6, and 9) presented characteristic mobilities (Fig. 1E), and the appearance of distinct bands revealed evidence of TAPBPR/MHC-I complex formation. Using this assay, we observed complex formation between hTAPBPR and HLA-A*02:01 (lane 4) and, to a lesser extent, with HLA-A*24:02 (lane 5), but not with HLA-A*01:01 (lane 13) (Fig. 1E). However, moTAPBPR formed a complex only with HLA-A*02:01 (lane 7), and chTAPBPR solely interacted with HLA-A*24:02 (lane 11), showing no binding to HLA-A*01:01 (lanes 14 and 15) under our experimental conditions (Fig. 1E). These results suggest that recombinant TAPBPR proteins have distinct HLA-binding profiles.

### TAPBPR orthologs exhibit distinct binding profiles with HLAs captured on single antigen beads

**Fig. 2.**
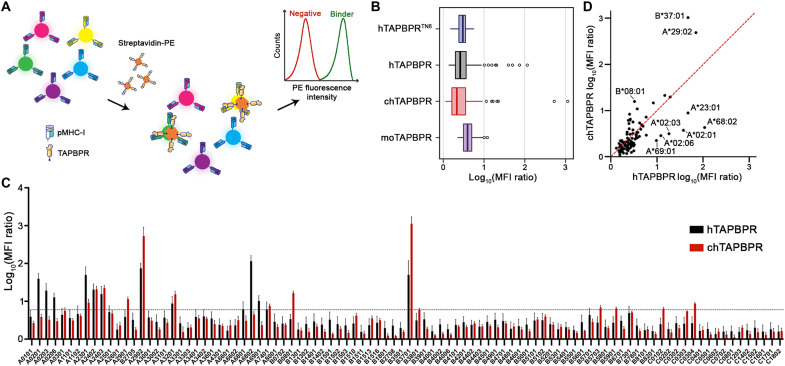
TAPBPR orthologs demonstrate distinct HLA-binding profiles using SABs. (**A**) Schematic representation of the SAB assay used to measure the levels of binding to individual HLA-I allotypes by streptavidin–phycoerythrin (PE)–conjugated TAPBPR orthologs. SABs are color-coded and are decorated with different HLA-I allotypes. The SABs were incubated with 7 μM tetramerized TAPBPR for 1 hour at room temperature (RT). (**B**) Box plot showing the distribution of the TAPBPR MFI ratios for the 96 HLA-I allotypes. hTAPBPR^TN6^ was used to determine interactions between TAPBPR orthologs and MHC-I molecules. The boundary of the box closest to zero indicates the 25th percentile, the line within the box marks the median, and the boundary of the box farthest from zero indicates the 75th percentile. Whiskers above and below the box indicate the 10th and 90th percentiles. The points above the whiskers indicate outliers outside the 90th percentile. (**C**) Bar graph showing the levels of hTAPBPR and chTAPBPR binding to the SABs. Incubation with the W6/32 antibody before tetramer staining was used to control for the nonspecific background binding, and the MFI ratio of TAPBPR/control was determined. A cutoff of log_10_ of 5.97 (0.77) was set to determine an interaction as significant, calculated from the mean MFI ratio of hTAPBPR^TN6^ + 3σ. Plotted data are means ± σ from three independent experiments. (**D**) Correlation of hTAPBPR and chTAPBPR log_10_ (MFI ratio) plotted in (C). The dashed red line represents a conceptual 1:1 correlation (no difference between orthologs).

To evaluate interactions between recombinantly expressed TAPBPR from different species and a broad range of human MHC-I allotypes, we used HLA class I single antigen beads (SABs). The beads are fluorescently color-coded and coated with 96 different HLA allotypes (*38*, *39*), derived from expression in Epstein-Barr virus (EBV)–transformed cell lines and thus loaded with a “garden variety” of cell-derived peptides. An SAB-based assay has been previously applied to study interactions between hTAPBPR and HLA-I (*22*). Here, we further adapted the method to capture interactions of different TAPBPR orthologs by creating multivalent tetramers (Fig. 2A), as previously described (*40*). The advantages of using tetramers are to improve avidity for identifying low-affinity interactions with MHC-I and allowing direct comparisons of MHC-I binding across TAPBPR orthologs, bypassing primary anti-TAPBPR antibodies.

To confirm the conformational integrity of HLA-I molecules, we compared the staining levels using the pan-HLA class I reactive monoclonal antibody W6/32, which recognizes a conformational epitope on heavy chain/β_2_m complexes (*41*), with contributions from both polypeptide chains (*42*). Mean fluorescence intensity (MFI) levels were similar for all beads, revealing homogeneous protein levels across all HLA-I allotypes (fig. S3A). Since the TAPBPR binding epitope on MHC-I is masked by W6/32 binding, we preincubated the beads with W6/32, followed by tetramer addition to control for nonspecific background staining levels. In agreement, the MFI levels of bound TAPBPR tetramers were substantially reduced upon incubation with W6/32 (fig. S3, B to E). Thus, we used the MFI ratio of TAPBPR tetramer staining signal relative to this control experiment to quantify interactions with different HLA allotypes. In addition, we used the TN6 mutant of TAPBPR (hTAPBPR^TN6^: E205K, R207E, Q209S, and Q272S) (Fig. 2B and figs. S2B and S3B), which does not interact with HLA-A*02:01 (*10*, *11*), to further control for nonspecific binding by choosing a threshold of 5.97 (the mean MFI ratio of hTAPBPR^TN6^ + 3σ) to define significant interactions, relative to the control (Fig. 2C).

Analysis of MFI ratios revealed substantial differences in interaction profiles exhibited by hTAPBPR and chTAPBPR with different HLA supertype representatives. In particular, hTAPBPR showed high levels of binding to HLA-A*32:01 (A01 supertype); HLA-A*29:02 (A01/A24 supertype); HLA-A*02:01, HLA-A*02:03, HLA-A*02:06, HLA-A*68:02, and HLA-A*69:01 (A02 supertype); HLA-A*68:01 and HLA-A*74:01 (A03 supertype); HLA-A*23:01, HLA-A*24:02, and HLA-A*24:03 (A24 supertype); and HLA-B*37:01 (B44 supertype) (Fig. 2C and table S1). chTAPBPR showed high levels of binding to HLA-A*32:01 (A01 supertype); HLA-A*29:01 and HLA-A*29:02 (A01/A24 supertype); HLA-A*74:01 (A03 supertype); HLA-A*23:01, HLA-A*24:02, and HLA-A*24:03 (A24 supertype); HLA-B*08:01 (B08 supertype); HLA-B*37:01 (B44 supertype); HLA-B*57:03 (B58 supertype); HLA-B*59:01 (unclassified); and HLA-C*01:02 and HLA-C*03:04 (C01 supertype), thereby demonstrating differential binding to HLA representatives and covering complementary sets of HLA-B and HLA-C supertypes (Fig. 2, C and D, and table S1). Conversely, analogous SAB experiments using the mouse ortholog showed MFI ratios less than 5.97, suggesting weak interactions across HLA allotypes (fig. S3E). In summary, our high-throughput SAB assay demonstrates that different TAPBPR orthologs exhibit distinct interaction profiles with HLA-I allotypes, in agreement with our preliminary EMSA analysis (Fig. 1E).

### Differential recognition of peptide-deficient relative to loaded MHC-I molecules by hTAPBPR and chTAPBPR

**Fig. 3.**
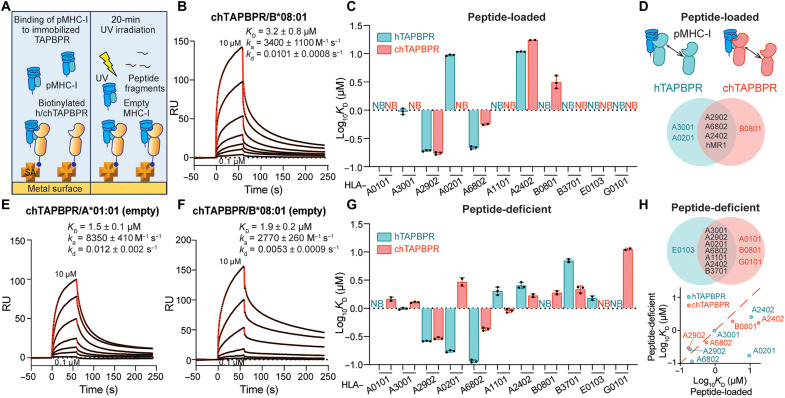
TAPBPR orthologs recognize peptide-deficient HLA molecules with a broad allelic specificity in vitro. (**A**) Schematic diagram showing TAPBPR orthologs immobilized on the SPR sensor chip. Different photolabile pHLA molecules at various concentrations with or without UV irradiation flow over the surface. (**B**) Representative sensorgram of HLA-B*08:01/FLRGRAJGL flowed over a streptavidin chip coupled with chTAPBPR-biotin. (**C**) Log-scale comparison of *K*_D_ values for chTAPBPR or hTAPBPR interacting with HLA allotypes as indicated. (**D**) Schematic summary of pHLA molecules interacting with hTAPBPR or chTAPBPR in light blue or red, respectively. Allotypes that bind to hTAPBPR, chTAPBPR, or both are colored in blue, red, and black. (**E** and **F**) Representative sensorgrams of UV-irradiated HLA-A*01:01 (E) and HLA-B*08:01 (F). (**G**) Log-scale comparison of *K*_D_ values for chTAPBPR or hTAPBPR interacting with peptide-deficient HLA allotypes as indicated. (**H**) Schematic summary of peptide-deficient HLA molecules interacting with hTAPBPR or chTAPBPR in light blue or red, respectively. Allotypes that bind to hTAPBPR, chTAPBPR, or both are colored in blue, red, and black. Correlation of peptide-loaded and -deficient HLA binding to hTAPBPR and chTAPBPR log_10_ *K*_D_ plotted in (C) and (D). The dashed red line represents a conceptual 1:1 correlation (no difference between peptide-loaded and -deficient molecules). *K*_D_, equilibrium constant; *k*_a_, association rate constant; *k*_d_, dissociation rate constant; RU, resonance units. The concentrations of analyte for the top and the bottom sensorgrams are noted. Fits from the kinetic analysis are shown with red lines. Results of at least two technical replicates (means ± σ) are plotted.

To further explore interactions between TAPBPR orthologs and different HLA allotypes in a peptide-dependent manner, we used surface plasmon resonance (SPR) as a quantitative assessment of protein-protein interactions (Fig. 3A). A set of recombinant HLA allotypes covering all six HLA-A supertypes and two HLA-B supertypes (*33*) were refolded with hβ_2_m and photolabile peptides (*43*). We confirmed that peptide-loaded HLA (pHLA)–B*08:01 interacted with chTAPBPR, but not hTAPBPR, with a low micromolar–range dissociation equilibrium constant (*K*_D_), in agreement with our SAB assay results (Fig. 3, B and C, and fig. S5A). pHLA-A*24:02 and pHLA-A*68:02 exhibited higher binding affinities toward hTAPBPR relative to chTAPBPR (Fig. 3C, and fig. S4, E and G). pHLA-A*29:02 and pHLA-A*68:02 bound to both orthologs with nanomolar-range *K*_D_ values and slow dissociation rate constants (*k*_d_) (Fig. 3C, and fig. S4, C and E). pHLA-A*02:01 and pHLA-A*30:01 interacted with hTAPBPR but not chTAPBPR (Fig. 3C, and fig. S4, B and D). As established previously for hTAPBPR (*11*, *22*, *27*), pHLA-A*01:01 showed no interaction with either ortholog (Fig. 3C and fig. S4A). Notably, pHLA-B*37:01 showed no direct interactions with both chaperones (Fig. 3C and fig. S5B), which seemed to contradict our SAB results. Here, one important caveat is that TAPBPR binding to MHC-I molecules is highly dependent on groove peptide occupancy (*11*, *23*, *25*, *44*) and demonstrates a narrow specificity toward peptide-loaded MHC-I with micromolar-range affinities (Fig. 3D).

We further investigated interactions between TAPBPR orthologs and empty HLA proteins by performing a 20-min UV irradiation on these photolabile peptide–loaded molecules before SPR (Fig. 3A) (*11*, *44*). We found that, while HLA-A*01:01 did not bind to hTAPBPR in agreement with previous reports (*11*), empty molecules demonstrated a low micromolar–range binding to chTAPBPR (Fig. 3E and fig. S4A). Compared to pHLA-B*08:01, empty HLA-B*08:01 showed tighter binding to chTAPBPR due to a twofold decreased *k*_d_ (Fig. 3, B and F, and fig. S5A). Peptide-deficient HLA-A*68:02 and HLA-A*24:02 interacted with hTAPBPR and chTAPBPR with higher affinities relative to their peptide-loaded states (Fig. 3, C and G, and fig. S4, E and G). Furthermore, both orthologs bound to empty HLA-A*11:01 and HLA-B*37:01, but not to the respective peptide-loaded molecule (Fig. 3G and figs. S4F and S5B). Similar to HLA-A*02:01, HLA-A*30:01 showed direct binding to chTAPBPR upon UV irradiation (Fig. 3G, and fig. S4, B and D). HLA-A*29:02 in either peptide-loaded or -deficient states demonstrated a low nanomolar–range interaction with both orthologs (Fig. 3G and fig. S4C). While empty HLA-A*30:01, HLA-A*29:02, HLA-A*02:01, and HLA-A*68:02 exhibited tighter binding to hTAPBPR, empty HLA-A*11:01, HLA-A*24:02, and HLA-B*37:01 showed higher affinities toward chTAPBPR (Fig. 3G). In summary, peptide-deficient HLA allotypes showed direct binding to at least one TAPBPR ortholog with higher affinities compared to peptide-loaded molecules (Fig. 3H). While consistent with our EMSA results, which showed that both orthologs demonstrated binding to HLA-A*24:02 (Fig. 1E), SPR further revealed interactions between chTAPBPR and empty HLA-A*02:01 with an almost doubled *k*_d_ compared to chTAPBPR/HLA-A*24:02. The lack of binding to empty HLA-A*02:01 in our native gel assay can be explained by instability of the complexes under our experimental conditions. The in vitro SPR and SAB assay were also reconciled, since SABs contained MHC-I molecules comprising a mixture of high- and intermediate-affinity peptides, thereby preventing a detailed analysis of the peptide dependence on interactions with molecular chaperones.

We then extended our study of TAPBPR interactions to cover the oligomorphic class Ib and nonclassical MHC-I molecules, namely, HLA-E*01:03, HLA-G*01:01, and MR1 C262S (*44*). We showed that acetyl-6-formylpterin–loaded MR1 C262S bound to hTAPBPR with moderate affinity, while the interaction with chTAPBPR was fivefold tighter (99 μM versus 19 μM; fig. S5E). The class Ib HLA allotypes tested showed no interaction with either hTAPBPR or chTAPBPR when prepared as peptide-loaded molecules. However, upon UV irradiation, peptide-deficient HLA-E*01:03 interacted with hTAPBPR, while HLA-G*01:01 interacted with chTAPBPR with moderate micromolar–range *K*_D_ (Fig. 3, C and G, and fig. S5, C and D). Together, our SPR data provide additional evidence that different TAPBPR orthologs can interact with complementary sets of HLA allotypes. Peptide-deficient MHC-I molecules generally exhibit tighter interactions with chaperones, while, in some cases, both TAPBPR orthologs recognize the empty MHC-I state exclusively, in agreement with previous studies (*11*, *27*).

### Design of conditional ligands to monitor peptide exchange across different HLA allotypes

To investigate the effects of interactions with TAPBPR orthologs on peptide exchange function for different HLA allotypes, we followed the binding of fluorescently labeled high-affinity peptides by real-time fluorescence polarization (FP) (*45*). Here, high-affinity fluorescent peptide probes exchange for placeholder peptides that have been preloaded on the HLA molecule of interest. We first identified suitable conditional ligands that served as placeholder peptides for various HLA allotypes. Previous approaches for generating these ligands use either photosensitive peptides treated with UV irradiation (*12*, *36*, *37*, *46*) or “Goldilocks” peptides derived from high-affinity peptide epitopes by truncation of the N-terminal amino acid (*25*, *47*). However, both approaches can lead to notable levels of background exchange in the absence of chaperones, an effect that can mask any additional exchange promoted by chaperones.

**Fig. 4.**
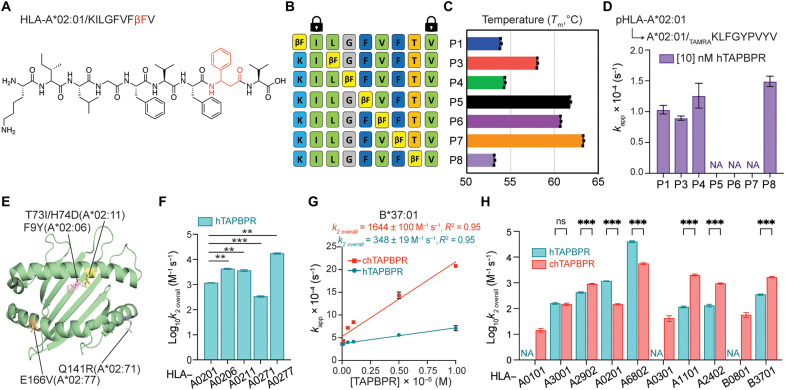
Conditional peptide ligands reveal the effects of HLA micropolymorphisms and supertype dependence of peptide exchange by TAPBPR orthologs. (**A**) Conditional peptide ligand KILGFVFβFV based on the HLA-A*02:01–restricted influenza matrix 1 (*58*–*65*, *78*) epitope GILGFVFTL. T8 is replaced with βF. (**B**) HLA-A*02:01 βF scanning peptide panel at each position as indicated. (**C**) *T*_m_ (in degrees Celsius) values obtained from DSF of HLA-A*02:01 bound to KILGFVFTV with βF substitution at indicated positions. (**D**) Comparison of *k*_app_ for fluorescent peptide binding to HLA-A*02:01/KILGFVFTV with βF substitution at indicated positions in the presence of 10 nM hTAPBPR. (**E**) Crystal structure of HLA-A*02:01 (*79*) (PDB ID: 3MRE) indicates the polymorphic residues for HLA-A*02:06 (light pink), HLA-A*02:11 (light yellow), HLA-A*02:71 (light blue), and HLA-A*02:77 (light orange). (**F**) Log-scale comparison of *k*_2overall_ for HLA-A*02:01, HLA-A*02:06, HLA-A*02:11, HLA-A*02:71, and HLA-A*02:77 in the presence of hTAPBPR. The two-sample unequal variance Student’s *t* test was performed, *P* > 0.12 (ns), *P < 0.033, **P < 0.002, and ***P < 0.001. (**G**) Linear correlations between the apparent rate constants *k*_app_ and the concentrations of TAPBPR orthologs for HLA-B*37:01. (**H**) Log-scale comparison of *k*_2overall_ for HLA-A*01:01, HLA-A*30:01, HLA-A*29:02, HLA-A*02:01, HLA-A*68:02, HLA-A*03:01, HLA-A*11:01, HLA-A*24:02, HLA-B*08:01, and HLA-B*37:01 in the presence of hTAPBPR or chTAPBPR. The apparent rate constant *k*_app_ was determined by fitting the raw trace to a monoexponential association model. The extrapolation of the slope between *k*_app_ and the concentrations of TAPBPR orthologs determines the overall rate *k*_2overall_. NA indicates no *k*_2overall_ (no peptide exchange) determined. Results of three technical replicates (means ± σ) are plotted.

Here, we used an alternative method to generate conditional ligands by introducing an unnatural amino acid l-β-phenylalanine (βF), structurally akin to the photolabile amino acid 3-amino-3-(2-nitrophenyl)-propionic acid (J-amino acid) (*37*) but without peptide cleavage under the UV exploration (Fig. 4A). We explored all single-residue substitutions of the epitope KILGFVFTV with βF on the HLA-A*02:01 system (Fig. 4B). We found that βF modifications at positions P5, P6, and P7 had a minimal effect on peptide stability, yielding refolded HLA-A*02:01 proteins with *T*_m_ values of 62°, 61°, and 63°C, respectively, which were indicative of high-affinity pMHC-I complexes (Fig. 4C). However, the introduction of βF at either P1, P4, or P8 had a substantial destabilization effect, yielding complexes with thermal stabilities in the range from 53°C (βF-P8) to 58°C (βF-P3) (Fig. 4C). This effect could be attributed to both the short, bulky phenyl ring side chain in sterically confined regions of the pMHC-I groove and the extra carbon-carbon bond in the peptide backbone that may introduce conformational “strain” to further destabilize the pMHC-I interaction compared to the conventional phenylalanine (*48*, *49*). We then monitored the exchange of each βF-containing conditional ligand on the HLA-A*02:01 groove for a fluorophore-conjugated, high-affinity peptide in the presence or absence of 10 nM hTAPBPR (*50*). We found that the effectiveness of hTAPBPR to catalyze the exchange of different placeholder peptides inversely correlated with the thermal stabilities of the βF peptide/HLA-A*02:01 complexes, where βF-P3 showed the smallest and βF-P8 the largest effect upon incubation with the chaperone (Fig. 4, C and D). Moreover, hTAPBPR at a concentration of 10 nM only accelerated the rate of reaction for βF peptides that already demonstrated a baseline level of exchange, whereas βF-P5, βF-P6, and βF-P7 showed a *T*_m_ value greater than 60°C and no peptide exchange either in the absence or presence of 10 nM hTAPBPR (Fig. 4D and fig. S6A). Since hTAPBPR recognizes peptide-deficient HLA-A*02:01 molecules with low nanomolar–range *K*_D_ as opposed to micromolar range for peptide-loaded molecules, a catalytic amount of hTAPBPR can stabilize empty MHC-I in a peptide-receptive conformation for peptide loading, in agreement with previous studies (*25*, *50*).

Furthermore, we monitored the exchange of the βF positional scanning peptides for HLA-A*02:01 with 5000 nM hTAPBPR. While hTAPBPR at a high concentration consistently promoted complete fluorescent peptide exchange for βF-P1, βF-P3, βF-P4, and βF-P8, it allowed peptide loading for βF-P5, βF-P6, and βF-P7, where no exchange was observed previously in the absence or presence of 10 nM hTAPBPR (fig. S6A). Expectedly, peptide exchange efficiency [apparent association rate constant (*k*_app_)] was substantially enhanced in a molar excess of hTAPBPR (Fig. 4D and fig. S6B). Therefore, hTAPBPR, at a molar excess, could recognize and chaperone the peptide-loaded molecules to unload their cargos and make empty molecules more accessible for the new incoming peptide. On the basis of the above results, we selected the βF-P8 peptide as the most suitable conditional ligand to investigate the effects of different TAPBPR orthologs on peptide exchange for HLA-A*02:01. The peptide exchange reaction of βF-P8–loaded HLA-A*02:01 was most effectively promoted under both a catalytic and molar excess amount of hTAPBPR (Fig. 4D and fig. S6B). We then used a similar strategy to design βF-substituted peptides for HLA-A*03:01, HLA-A*11:01, HLA-A*30:01, HLA-A*68:02, and HLA-B*37:01 (KLIETYFβFK, KTFPPTEβFK, ETAGIGILβFV, and FEDLRVβFSF) starting from the sequences of well-described immunodominant epitopes (fig. S6C). Using a combination of βF designs, destabilized peptides with J-amino acid (*51*) or other epitopic peptides (table S2), we obtained conditional pMHC-I molecules with optimal thermal stabilities (>50°C) to study chaperone-mediated exchange for representative HLA allotypes covering all six HLA-A supertypes and two HLA-B supertypes, in correspondence to our SPR-binding studies.

### TAPBPR orthologs catalyze peptide exchange on a broad repertoire of HLA supertypes

To quantitatively compare the effects of different TAPBPR orthologs on peptide exchange kinetics across different HLA allotypes, we performed a series of FP experiments in which pHLA molecules and fluorescent peptide probes at a fixed concentration were incubated with graded concentrations of the chaperone. The observed apparent association rate constants (*k*_app_) were determined by fitting a one-phase association equation to describe the fluorescent peptide ligand and MHC-I association. The slope extrapolated from the linear fit between *k*_app_ and TAPBPR concentration was defined as the overall catalytic rate (*k*_2overall_), which is analogous to the *k*_cat_ constant typically used to describe enzymatic reactions under steady-state conditions (*45*, *52*).

Previous studies have demonstrated that a small number of sequence variations within HLA subtypes, depending on their location, can control epitope length and influence TCR recognition (*53*, *54*). Single polymorphisms between different alleles within the HLA-B7 superfamily can induce a conformational change of identical HIV-1 peptides, which is associated with viral escape and disease progression (*55*). Here, we hypothesized that these differences among micropolymorphic HLA-A*02 allotypes could also affect TAPBPR recognition and peptide exchange kinetics. We found that hTAPBPR accelerated peptide exchange on the HLA-A*02:06, HLA-A*02:11, HLA-A*02:71, and HLA-A*02:77 subtypes at similar levels relative to HLA-A*02:01. However, for HLA-A*02:71 containing the Q141R polymorphism in the α_2–1_ helix, we observed a reduced *k*_2overall_ (335 M^−1^ s^−1^) by 3.5-fold compared to HLA-A*02:01 (Fig. 4, E and F, and fig. S7). Notably, for HLA-A*02:77, which contained an amino acid substitution on the opposite side of the α_2_ helix (E166V), we observed a significantly increased rate of peptide exchange relative to HLA-A*02:01 (17,185 M^−1^ s^−1^ versus 1162 M^−1^ s^−1^) (fig. S7). Given that both protein complexes with the same placeholder peptide exhibited a similar overall stability (table S3), the presence of allosteric communication across the α_2_ helix might also influence the MHC-I interactions with TAPBPR (*27*). Together, our results provide evidence that the interplay of polymorphic residues, both at the HLA-I/TAPBPR interface and removed from it, can fine-tune interactions and peptide exchange function.

We next examined the effects of both TAPBPR orthologs on peptide exchange across our panel of HLA supertype representatives. Analysis of our FP data revealed that, while the *k*_2overall_ of HLA-A*02:01 was eightfold higher for hTAPBPR than chTAPBPR, the *k*_*2*overall_ of B*37:01 in the presence of hTAPBPR was approximately fivefold lower than chTAPBPR (Fig. 4, G and H, and figs. S8C and S9B). Likewise, the overall exchange rate of HLA-A*11:01 and HLA-A*24:02 with the addition of chTAPBPR was more than 17- and 7-fold higher than hTAPBPR (Fig. 4H, and fig. S8, E and F). Placed within the context of our SPR data (Fig. 3, C and G), these results suggest that both orthologs promote peptide exchange by recognizing empty HLA allotypes with different affinities. In agreement, HLA-A*02:01 and HLA-A*68:02 with a substantially higher *k*_*2*overall_ for hTAPBPR also showed a higher affinity to hTAPBPR over chTAPBPR (0.17 μM versus 2.96 μM for HLA-A*02:01 and 0.12 μM versus 0.43 μM for HLA-A*68:02), and vice versa for HLA-A*11:01 (2.04 μM versus 0.91 μM), HLA-A*24:02 (2.54 μM versus 1.68 μM), and HLA-B*37:01 (7.02 μM versus 2.19 μM) (Figs. 3G and 4H and fig. S8). hTAPBPR and chTAPBPR catalyzed peptide exchange for HLA-A*30:01 or HLA-A*29:02 at a similar rate with less than onefold change of *k*_*2*overall_, demonstrating comparable affinities to the empty molecules (0.99 μM versus 1.28 μM for HLA-A*02:01 and 0.26 μM versus 0.29 μM for HLA-A*29:02) (Figs. 3G and 4H, and fig. S8, B and H). hTAPBPR showed no effect on peptide exchange for HLA-A*01:01, HLA-A*03:01, and HLA-B*08:01, whereas chTAPBPR effectively catalyzed the peptide loading for the same systems (Fig. 4H and figs. S8, A and D, and S9A). Therefore, our FP results covering all six HLA-A supertypes and two HLA-B supertypes (Table 1) support that TAPBPR catalysis proceeds through the recognition and stabilization of an empty MHC-I at a peptide-receptive conformation.

**Table 1.** A summary of the binding affinities to TAPBPR orthologs and the peptide exchange results for 13 MHC-I representatives.

	Supertype	A01	A01 A03	A01 A24	A02		A03		A24	B08	B44			
	Allele	HLA-A*								HLA-B*		HLA-E*	HLA-G*	
		01:01	30:01	29:02	02:01	68:02	03:01	11:01	24:02	08:01	37:01	01:03	01:01	MR1
Global population frequency (%)		13.2	2.0	2.6	24.1	1.3	12.3	6.3	9.6	8.4	1.3	36.1	74.0	
hTAPBPR *K*_D_* (μM)	Loaded	NB†	0.95	0.19	9.44	0.22	n.d.‡	NB†	10.9	NB†	NB†	NB†	NB†	98.7
	Empty	NB†	0.99	0.26	0.17	0.12	n.d.‡	2.04	2.54	NB†	7.02	1.51	NB†	
chTAPBPR *K*_D_* (μM)	Loaded	NB†	NB†	0.17	NB†	0.56	n.d.‡	NB†	17.4	3.19	NB†	NB†	NB†	19.2
	Empty	1.46	1.28	0.29	2.96	0.43	n.d.‡	0.91	1.68	1.89	2.19	NB†	11.22	
Peptide exchange§	hTAPBPR	No	Yes	Yes	Yes	Yes	Yes (*12*)	Yes	Yes	No	Yes			Yes (*44*)
	chTAPBPR	Yes	Yes	Yes	Yes	Yes	Yes	Yes	Yes	Yes	Yes			

*K_D_ measured by SPR.

†NB, no binding.

‡n.d., *K*_D_ not determined by SPR.

§On the basis of FP and/or DSF assays.

To further test this hypothesis and to confirm whether chTAPBPR enables gain-of-function peptide exchange, we focused on HLA-A*01:01 and HLA-B*08:01, both of which are not amenable to hTAPBPR binding and peptide exchange in vitro (*22*, *27*). We first selected high-affinity peptides with increased thermal stabilities by at least 5° relative to complexes refolded with suitable placeholder peptides for both systems (fig. S10, A and C). As expected, HLA-A*01:01 refolded with a moderate-affinity RAS self-epitope did not exchange in the presence of 10-fold molar excess of high-affinity peptides either spontaneously or upon incubation with hTAPBPR (fig. S10B), consistent with the observed lack of binding between hTAPBPR and HLA-A*01:01 in either peptide-loaded or -deficient states by SPR (Fig. 3, C and G). Notably, peptide exchange readily occurred when incubating pHLA-A*01:01 with chTAPBPR in the presence of different high-affinity epitopic peptides, leading to a more than 10° thermal shift (fig. S10B). The minor destabilization effect observed for chTAPBPR upon addition of a nonspecific peptide (fig. S10B) likely resulted from the formation of pMHC-I/chTAPBPR complexes with destabilized peptide-binding grooves (*27*). HLA-B*08:01 showed a partial thermal shift upon incubation with excess high-affinity peptide, revealing a spontaneous, partial peptide-loading effect mediated by empty molecules, which was not affected by the addition of hTAPBPR (fig. S10D). We found that a catalytic amount of chTAPBPR promoted the complete exchange of placeholder for different high-affinity peptides, as indicated by a single, left-shifted *T*_m_ peak, while the nonspecific peptide control failed to bind in the presence of either hTAPBPR or chTAPBPR (fig. S10D). Thus, our DSF assay results demonstrate that chTAPBPR, but not hTAPBPR, can promote the complete peptide exchange for HLA-A*01:01 and HLA-B*08:01 (Table 1). HLA-A*02:01 and HLA-B*37:01, which demonstrate high-affinity binding to both orthologs (Fig. 3G), undergo complete peptide exchange as opposed to the negative control in the presence of both orthologs (fig. S10, E to H). Together, our data further support a mechanism where TAPBPR orthologs, unless in large molar excess, primarily accelerate peptide exchange by capturing and maintaining empty MHC-I molecules in their peptide-receptive, open conformation.

### TAPBPR orthologs recognize conserved surfaces on empty MHC-I molecules

We next sought to investigate the recognition mechanism of MHC-I molecules by chTAPBPR. First, we established thermodynamic parameters describing the binding process between pHLA-B*08:01 and chTAPBPR using isothermal titration calorimetry (ITC) (*56*). ITC titrations of chTAPBPR were performed in the presence of threefold molar excess (450 μM) of the high-affinity peptide to ensure saturation of the peptide-binding groove. Our ITC data revealed that pHLA-B*08:01 exhibited a strong endothermic binding interaction with chTAPBPR (fig. S11A), similar to previous ITC results reporting on interactions between P18-I10/H2-D^d^ and P29/H2-L^d^ with hTAPBPR (*27*). Fitting of the sigmoidal isotherms using a one-site binding model yielded an apparent *K*_D_ of 4.6 μM, comparable to the 3.2 μM from our SPR measurements (Fig. 3B). We then isolated empty HLA-B*08:01/chTAPBPR complexes by incubating UV-irradiated HLA-B*08:01 loaded with FLRGRAJGL with chTAPBPR in a 1.5:1 stoichiometric ratio, followed by SEC (fig. S11B). Purified chTAPBPR/HLA-B08:01 complexes were receptive to high-affinity peptides, as evidenced by the appearance of discrete pMHC-I bands in an EMSA upon incubation with the high-affinity peptides EBV, T1D30, and T1D95, but not with a nonspecific HIV epitopic peptide (fig. S11C). Together, we demonstrate that chTAPBPR can recognize both peptide-loaded and -deficient molecules, albeit with different affinities, and maintain empty HLA-B*08:01 in a peptide-receptive state in a similar manner established using hTAPBPR and HLA-A*02:01 molecules (*11*, *50*).

**Fig. 5.**
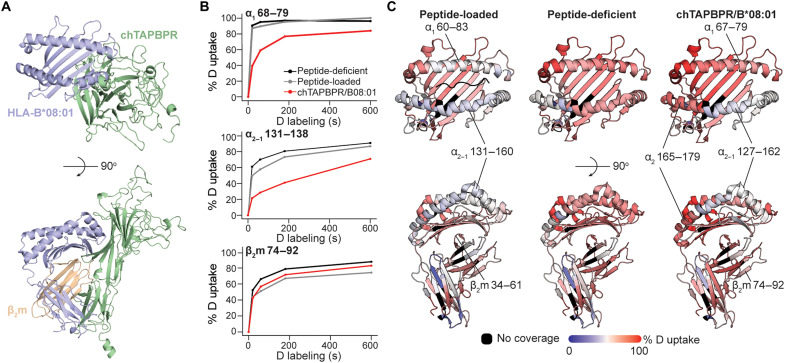
The chTAPBPR ortholog stabilizes HLA-B*08:01 in a receptive conformation by recognizing conserved epitopes on MHC-I relative to hTAPBPR. (**A**) A structural model of the HLA-B*08:01/chTAPBPR complex generated by RosettaCM (*72*) (BAKER-ROBETTA server). (**B**) The percent deuterium uptake resolved to individual peptide segments showing α_1_ 68 to 79, α_2–1_ 131 to 138, and β_2_m 74 to 92 is plotted for each exposure time (0, 20, 60, 180, and 600 s) and protein states. The plots reveal the local HDX profiles of HLA-B*08:01 for the states of peptide-deficient (black; UV irradiation for 40 min at 4°C), peptide-loaded (gray; refolded with FLRGRAJGL), and chTAPBPR-bound complex (red; UV irradiated for 40 min at RT in the presence of excess chTAPBPR, followed by SEC purification of the complex peak). (**C**) The percent deuterium uptake resolved to individual residues upon 60-s deuterium labeling for peptide-loaded (left), peptide-deficient (middle), and chTAPBPR-bound complex (right) states is mapped onto the HLA-B*08:01 crystal structure (*58*) (PDB ID: 4QRU) for visualization. Red- and blue-colored regions indicate segments containing peptides with 100% ΔHDX (red—more deuteration) or 0% ΔHDX (blue—less deuteration), respectively; black indicates regions where peptides were not obtained for peptide-loaded, peptide-deficient, and chTAPBPR-bound protein states.

To further probe chTAPBPR interactions and map the binding epitopes on HLA-B*08:01 in a solution environment, we performed hydrogen/deuterium exchange mass spectrometry (HDX-MS) (*57*). A structural model of the HLA-B*08:01/chTAPBPR complex, obtained using the murine MHC-I/hTAPBPR structure as a template (*24*), suggests potential interaction surfaces on the HLA-B*08:01 sequence (Fig. 5A). HLA-B*08:01, prepared as empty, peptide-bound, or chTAPBPR-bound, was incubated in a deuterated buffer for several time points, allowing for the incorporation of deuterium into the protein amide groups. The exchange reaction was quenched by a shift to acidic pH and digested by an acid-functional protease. Tandem analysis of the reaction products by MS identified three putative interaction regions with lower deuterium uptake when bound to chTAPBPR: residues 68 to 79 and 131 to 138 on the HLA-B*08:01 α_1_ and α_2–1_ regions and residues 74 to 92 on β_2_m (Fig. 5B). The percentage of deuterium uptake was mapped on an HLA-B*08:01 structure (PDB ID: 4QRU) (*58*), revealing binding sites on the α_1_ and α_2–1_ helices of the heavy chain and β_7_ of β_2_m (Fig. 5C), consistent with interaction sites observed in the structure of murine H2-D^d^ bound to hTAPBPR (PDB ID: 5WER). When comparing the peptide-loaded to the peptide-deficient state, deuterium uptake decreased in the peptide-binding domain, the membrane-proximal α_3_ domain, and the noncovalently associated β_2_m (Fig. 5C), highlighting a substantially reduced plasticity upon peptide loading consistent with the previous study (*59*). Deuterium uptake of chTAPBPR-bound HLA-B*08:01 relative to empty molecules reached a maximum at 60 s and then gradually converged toward more similar levels (Fig. 5B), indicating reversible TAPBPR interactions in further agreement with our SPR data showing a low micromolar–range *K*_D_ (Fig. 3G). Increased deuterium uptake at residues 165 to 179 on the α_2_ helix upon chTAPBPR binding provides evidence of long-range effects, namely, allosteric coupling between the F and B pockets in agreement with the previous studies (*50*, *60*, *61*). In summary, our HDX-MS mapping reveals that chTAPBPR recognizes common epitopes on MHC-I, as seen previously in both the cocrystal structures of murine MHC-I/hTAPBPR complexes (*23*, *24*) and solution NMR–based mapping of HLA-A*02:01/hTAPBPR complexes (*27*, *50*).

### TAPBPR sequence variants enable gain-of-function interactions with targeted HLA allotypes

To further understand how TAPBPR sequence variation affects interactions with HLAs, we used cell-based assays to explore thousands of substitutions in hTAPBPR-TM, a chimeric construct containing the luminal domain of hTAPBPR fused to the canonical transmembrane domain of HLA-G. Overexpression of hTAPBPR could partially rescue the surface expression of HLA-A*02:01 that was reduced by tapasin KO in human Expi293F cells, consistent with the previous report (fig. S12A) (*27*). However, overexpressing the conventional negative control hTAPBPR^TN6^, which is known to disrupt binding to mature, folded MHC-I (*11*), could also effectively rescue surface HLA-A*02:01 in a tapasin-KO background (fig. S12A). Since hTAPBPR and hTAPBPR^TN6^ were trapped in ER and Golgi during this assay (*2*), they both participated in intracellular chaperoning of nascent MHC-I. Alternatively, we used hTAPBPR-TM, which could be expressed within the ER and on the cell surface (*27*). An analogous construct to hTAPBPR-TM was previously found to associate with MHC-I on the cell surface to promote peptide editing (*62*). Therefore, not only did hTAPBPR-TM rescue HLA-A*02:01 surface expression in tapasin-KO Expi293F cells, but this effect was also now lost in the hTAPBPR-TM^TN6^ mutant. The hTAPBPR-TM–mediated rescue of surface HLA-A*02:01 is an indirect readout of the physical association between hTAPBPR and folded MHC-I rather than nascent molecules, an essential feature of peptide editing. On the basis of these observations, we used hTAPBPR-TM deep mutational scanning to identify hTAPBPR mutants with enhanced peptide-editing function for HLA-A*02:01.

A single site-saturation mutagenesis (SSM) library of hTAPBPR-TM was created, focusing on 75 residues in the hTAPBPR luminal domain that interface with MHC-I based on the crystal structures (fig. S13A). We also mutated 17 additional residues at control sites: (i) on the opposite side of hTAPBPR to the interface where mutations were expected to have a minimal effect (fig. S13C) and (ii) in the protein core where most mutations were expected to be highly deleterious (fig. S13B). The library was transfected into the tapasin-KO Expi293F line, and cells with high levels of surface HLA-A*02:01 were collected by fluorescence-activated cell sorting (FACS) (fig. S14). The naive and sorted libraries were deep-sequenced, and the enrichment of functional mutations or depletion of deleterious mutations was calculated to determine a mutational landscape. The data were closely correlated between two independent selection experiments, and mutations at control sites had the anticipated effects (fig. S13, E and F).

**Fig. 6.**
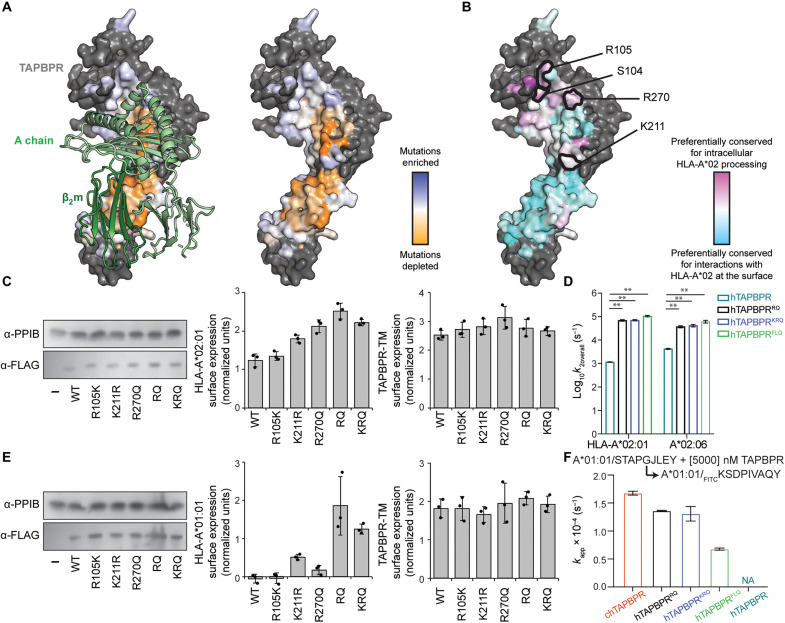
Deep mutational scanning of TAPBPR surface enhances peptide exchange function for HLA-A*02:01 and HLA-A*01:01. (**A**) Sequence conservation from deep mutagenesis is mapped to the surface of hTAPBPR (*24*) (PDB ID: 5WER, mouse H2-D^d^/hβ_2_m shown as light and dark green ribbons at left). Mutationally depleted or tolerant residues are colored orange or white and blue. Nonmutated residues are gray. (**B**) Conservation scores from the deep mutational scan of hTAPBPR-TM versus hTAPBPR (*50*). Residues preferentially conserved for intracellular processing or surface interaction of HLA-A*02:01 are pink or cyan. Positions S104, R105, K211, and R270 are identified as more conserved for intracellular HLA-A*02:01 processing and are sites of gain-of-function mutations for promoting surface HLA-A*02:01 trafficking. (**C**) Immunoblots comparing total expression levels for TAPBPR mutants (α-FLAG) versus PP1B as a loading control (left). Surface expression of HLA-A*02:01 in the presence of the different TAPBPR-TM mutants (middle). Surface expression for the different TAPBPR-TM mutants (right). (**D**) Log-scale comparison of *k*_2overall_ for HLA-A*02:01 and HLA-A*02:06 in the presence of hTAPBPR or mutants. The two-sample unequal variance Student’s *t* test was performed, *P* > 0.12 (ns), *P < 0.033, **P < 0.002, and ***P < 0.001. (**E**) Immunoblots comparing total expression levels for TAPBPR mutants (α-FLAG) versus PP1B as a loading control (left). Surface expression of HLA-A*01:01 in the presence of the different TAPBPR-TM mutants (middle). Surface expression for the different TAPBPR-TM mutants (right). (**F**) Comparison of *k*_app_ for fluorescent peptide binding to HLA-A*01:01 in the presence of 5 μM chTAPBPR, hTAPBPR mutants, and hTAPBPR. Results of three replicates (means ± σ) are plotted.

In the deep mutational scan, most hTAPBPR residues that contacted MHC-I and especially β_2_m, tended to be conserved and intolerant of mutations, consistent with the hTAPBPR-TM chimera engaging folded MHC-I at the cell surface with β_2_m interactions contributing to recognition (Fig. 6A). However, there were two prominent regions at the interface where mutational tolerance was high. The first region comprises the 24-35 loop that hovers above the F pocket of the peptide groove and acts as a “trap” to prevent high-affinity peptides from dissociating when TAPBPR forms transient associations with peptide-loaded MHC-I (fig. S13D) (*50*). The second interface region of mutational tolerance is formed by hTAPBPR residues that contact the MHC-I α_2_ helix (Fig. 6A). The same TAPBPR residues are mildly conserved for chaperoning function (*50*) (Fig. 6B), suggesting that this region may have different roles in chaperoning versus peptide editing.

We hypothesized that we could engineer an hTAPBPR-TM mutant with enhanced HLA-A*02:01 interactions by focusing substitutions to the two regions in proximity to MHC-I yet of high mutational tolerance in the deep mutational scan. We identified five highly enriched mutations, S104F, R105K, K211R, K211L, and R270Q, in the scan of hTAPBPR-TM for the rescue of HLA-A*02:01 surface expression, but not as enriched in the previously published mutational scan of intracellular hTAPBPR chaperoning HLA-A*02:01 (Fig. 6B), suggesting that these mutations may enhance interactions with folded molecules. Therefore, single and combination mutants at the selected positions of hTAPBPR-TM were tested for their ability to rescue HLA-A*02:01 surface expression in tapasin-KO cells. TAPBPR-TM mutants S104F and R105K mediated minor increases in HLA-A*02:01 surface expression that was not statistically significant, while TAPBPR-TM mutants containing individual or combined mutations at position R105, K211, and R270 had enhanced rescuing activity for surface HLA-A*02:01 compared to WT TAPBPR-TM (Fig. 6C and fig. S12B). We noted, however, that in this cell-based assay, chTAPBPR-TM and moTAPBPR-TM could not rescue surface HLA-A*02:01 levels (fig. S12A) possibly because both orthologous proteins were expressed at much higher levels intracellularly where they might bind and sequester MHC-I substrates.

We then purified recombinantly expressed proteins encompassing the soluble ectodomain of three hTAPBPR mutants carrying different combinations among five mutations: hTAPBPR^RQ^ (K211R and R270Q) and hTAPBPR^KRQ^ (R105K, K211R, and R270Q) carried substitutions to polar amino acids, while hTAPBPR^FLQ^ (S104F, K211L, and R270Q) carried two substitutions for hydrophobic amino acids (fig. S15). Using our FP peptide exchange assay, we found that the mutants were extraordinarily efficient at catalyzing the exchange of KILGFVFβFV-loaded HLA-A*02:01 with the high-affinity fluorescent peptide _TAMRA_KLFGYPVYV. The *k*_2overall_ of hTAPBPR^RQ^ and hTAPBPR^KRQ^ for the HLA-A*02:01 peptide–editing reaction was approximately 60-fold higher than for hTAPBPR (Fig. 6D and fig. S16A). Unexpectedly, the *k*_2overall_ of pHLA-A*02:01–loading fluorescent peptide in the presence of hTAPBPR^FLQ^ increased by 90-fold compared to hTAPBPR (Fig. 6D and fig. S16A). Consistent with the previous FP results that hTAPBPR effectively promoted peptide exchange on micropolymorphic HLA-A*02 subtypes (Fig. 4F), we demonstrated that all three mutants enhanced the peptide exchange kinetics of HLA-A*02:06 (Fig. 6D and fig. S16B). Both hTAPBPR^RQ^ and hTAPBPR^KRQ^ improved the *k*_2overall_ of pHLA-A*02:06 by approximately ninefold compared to hTAPBPR, while hTAPBPR^FLQ^ showed the most pronounced effect of a 14-fold increase among the mutants (fig. S16B). In addition, hTAPBPR^FLQ^ at a concentration of 10 nM promoted exchange on HLA-A*02:01 loaded with βF peptides at P1, P3, P4, and P8 more efficiently than hTAPBPR at the same concentration, as indicated by the enhanced *k*_app_ (Fig. 6D, and fig. S16, C and D). HLA-A*02:01/βF-P6, previously showing no peptide exchange in the presence or absence of 10 nM hTAPBPR, was exchanged for the fluorescent peptide with a fitted *k*_app_ by 10 nM hTAPBPR^FLQ^ (fig. S16C). HLA-A*02:01 proteins loaded with each of the seven βF peptides, including βF-P5 and βF-P7, were all observed for peptide exchange under 100 nM hTAPBPR^FLQ^ condition (fig. S16, C and D). Therefore, selected mutations not only can enhance the surface expression of HLA-A*02:01 in the cell-based assay but are also functionally active in vitro with improved catalysis of peptide exchange.

In addition, recombinant hTAPBPR did not interact with or catalyze peptide exchange on HLA-A*01:01 in vitro, and, likewise, overexpression of hTAPBPR-TM could not increase surface HLA-A*01:01 levels in a tapasin-KO Expi293F cell line stably transfected with HLA-A*01:01 (fig. S12, C and D). However, we hypothesized that hTAPBPR-TM mutants with enhanced activity for the rescue of surface HLA-A*02:01 in tapasin-KO cells might have gained activity toward HLA-A*01:01. hTAPBPR-TM carrying the substitutions K211R and/or R270Q, which were coincidentally aligned with the chTAPBPR sequence, stabilized HLA-A*01:01 at the cell surface (Fig. 6E). Furthermore, whereas purified soluble hTAPBPR had no effect on the exchange of a placeholder peptide on HLA-A*01:01 for a fluorescent peptide, the apparent rate constants *k*_app_ of hTAPBPR^RQ^ (1.4 × 10^−4^ s^−1^) and hTAPBPR^KRQ^ (1.3 × 10^−4^ s^−1^) at 5 μM were close to the *k*_app_ of chTAPBPR (1.7 × 10^−4^ s^−1^) on HLA-A*01:01 (Fig. 6F and fig. S16F). Recombinant hTAPBPR^FLQ^ also promoted peptide exchange on HLA-A*01:01 but was most inefficient among the mutants with a *k*_app_ of 0.7 × 10^−4^ s^−1^ at 5 μM (Fig. 6F fig. S16F). Unlike chTAPBPR, both triple mutants hTAPBPR^KRQ^ and hTAPBPR^FLQ^ failed to catalyze peptide exchange on HLA-B*08:01 (fig. S16E), demonstrating that the mutations do not universally enhance TAPBPR activity but are specific for a subset of MHC-I allotypes. Overall, our engineering efforts based on deep mutagenesis in a cell-based assay demonstrate how even a small number of mutations can manipulate the HLA allele specificity of TAPBPR.

### Conserved and polymorphic epitopes on MHC-I and TAPBPR determine allele-specific interactions

**Fig. 7.**
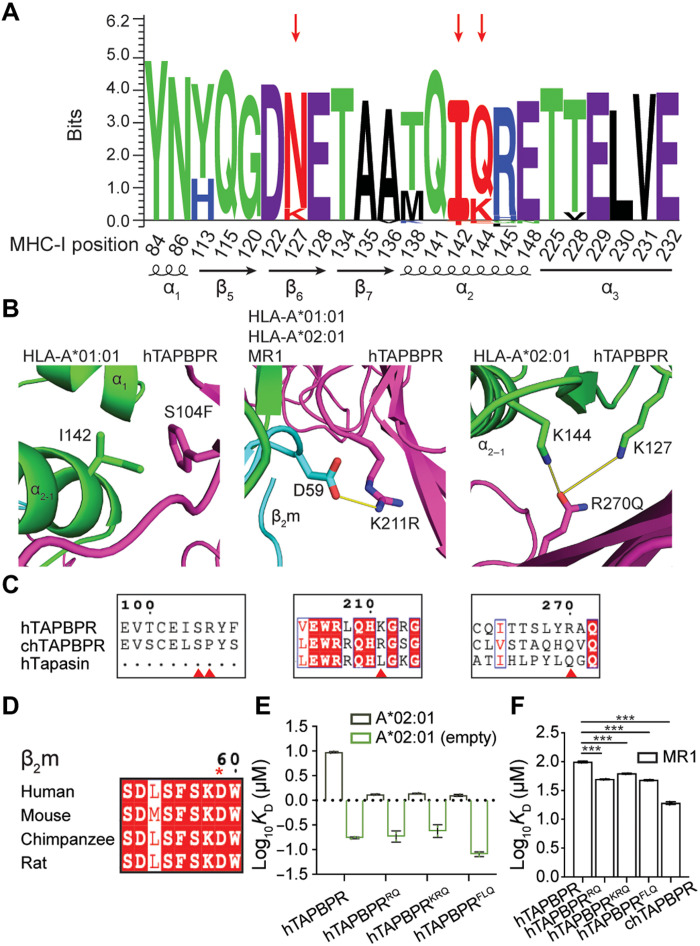
Targeted mutagenesis of hTAPBPR surfaces can promote interactions with conserved or polymorphic epitopes on classical and nonclassical MHC-I molecules. (**A**) Seq2logo (*80*) visualization of MHC-I residues that interact with TAPBPR calculated from the sequences of 75 different HLA allotypes. Sequence weighting used clustering, pseudo-count with a weight of 0, and Kullback-Leibler logotype. The percentage frequency of amino acids on a specific position higher than 10% is shown on the positive *y* axis and less than 10% amino acids on the negative *y* axis. (**B**) Structural models of HLA-A*01:01 or HLA-A*02:01 in complex with hTAPBPR generated by RosettaCM (*72*) (BAKER-ROBETTA server). Amino acids at positions 142 of HLA-A*01:01, 59 of hβ_2_m, and 127 and 144 of HLA-A*02:01 are indicated. (**C**) Sequence alignment of hTAPBPR, chTAPBPR, and human tapasin (hTapasin) near positions 104, 105, 211, and 270. (**D**) Sequence conservation patterns within the same region of β_2_m from the amino acid position 58 to 60 across four species. (**E**) Log-scale comparison of SPR determined *K*_D_ values for peptide-loaded and empty HLA-A*02:01 interacting with hTAPBPR and TAPBPR mutants. (**F**) Log-scale comparison of SPR determined *K*_D_ values for MR1 C262S interacting with hTAPBPR, TAPBPR mutants, and chTAPBPR. The two-sample unequal variance Student’s *t* test was performed, *P* > 0.12 (ns), *P < 0.033, **P < 0.002, and ***P < 0.001. Results of at least two technical replicates (means ± σ) are plotted.

To further understand the sequence and structural basis of how specific TAPBPR amino acid substitutions can promote interactions with HLA-A*02:01, HLA-A*01:01, and other relevant allotypes, we analyzed MHC-I polymorphic residues located on the TAPBPR interaction surfaces from 75 common (>1% population prevalence) HLA allotypes. We found a strong sequence conservation pattern of TAPBPR-interacting residues on the MHC-I with some variations at specific sites (Fig. 7A). The selection of HLA supertype representatives covering the polymorphisms within the peptide binding groove also sufficiently depicts the level of sequence variation within the TAPBPR-contacting residues of different HLA allotypes (table S4). We then performed structure-based modeling of the identified TAPBPR mutants using the x-ray structure of a mouse MHC-I/hTAPBPR complex (*24*) as a template, together with either HLA-A*01:01 or HLA-A*02:01. Inspection of the resulting models revealed that in hTAPBPR^FLQ^, S104F is located on the N-terminal immunoglobulin V (IgV) domain where it forms hydrophobic contacts with the outer rim of the MHC-I α_2–1_ helix, and specifically with I142 on HLA-A*01:01 (Fig. 7B). Within HLA-A and HLA-B alleles, position 142 is either I or T. In structural models of hTAPBPR^RQ^ and hTAPBPR^KRQ^, K211R is positioned at a surface that cradles the underside of the pMHC-I groove, including the nonclassical molecule MR1 (Fig. 7B). The side chain of R211 interacts with the conserved residue D59 within the 58-60 loop of β_2_m hypothesized to be involved in peptide sensing (Fig. 7, B and D) (*24*). Last, models generated using R270Q removed the positively charged residue and revealed possible polar interactions with the side chains from residues K127 and K144 in HLA-A*02:01 (Fig. 7B). A Q at position 270 of TAPBPR is also likely to stabilize complexes with HLA-A*01:01 or MR1, given that the corresponding residues are N127 and K144 in both molecules. HLA alleles have either a K or N at position 127 and a Q or K or E at position 144, suggesting a basis for the divergence of different alleles in their interactions with chaperones, which can be leveraged to design allele-specific catalysts, as exemplified in our study (Fig. 7A). The mutational tolerance of these hTAPBPR residues in the deep mutational scan may therefore partly reflect a lack of close structural complementarity to allow for recognition of polymorphic sites among HLA alleles. We note that the sequences of TAPBPR orthologs were not considered in selecting mutations, as our objective was to explore different routes to enhance the peptide editing function of TAPBPR. Nonetheless, a sequence alignment revealed that hTAPBPR amino acid substitutions K211R/L and R270Q were shared with chTAPBPR and human tapasin (Fig. 7C) that have preferential binding to HLA-B allotypes. Thus, structure modeling underscores how TAPBPR interactions with conserved and polymorphic residues of HLA molecules, including the β_2_m subunit, can be exploited to design gain-of-function variants, showing congruence with natural mutations that have occurred during evolution.

We have demonstrated that recombinant TAPBPR mutants can increase the *k*_2overall_ of HLA-A*02:01 peptide exchange by nearly two orders of magnitude relative to hTAPBPR, in agreement with our results from measuring TAPBPR-TM–mediated surface trafficking of HLA-A*02:01 in tapasin-KO cells, suggesting an increased binding affinity (Fig. 6, C and D). To quantitatively characterize this effect, we measured binding affinities of peptide-loaded or empty HLA-A*02:01 molecules to the three hTAPBPR mutants using SPR. pHLA-A*02:01 had a similar dissociation equilibrium constant, *K*_D_, toward all three TAPBPR mutants (Fig. 7E), which is approximately sevenfold tighter to hTAPBPR mutants than hTAPBPR (fig. S17A). All three mutants had similarly low nanomolar–range *K*_D_ values for empty HLA-A*02:01 (Fig. 7E). hTAPBPR^FLQ^, consistent with its remarkable performance on peptide exchange and tight binding toward pHLA-A*02:01, had the lowest *K*_D_ for peptide-deficient molecules (81 nM), compared to hTAPBPR^RQ^ (188 nM) and hTAPBPR^KRQ^ (243 nM) (fig. S17B). Last, we evaluated TAPBPR interactions with the nonclassical MHC molecule MR1 C262S, which have been shown to catalyze the exchange of noncovalent metabolite ligands (*44*). While chTAPBPR bound MR1 C262S fivefold tighter than hTAPBPR (19 μM versus 99 μM, respectively), the *K*_D_ values of the TAPBPR mutants were reduced by approximately twofold (Fig. 7F and fig. S17C). Likely because of the S104F and R270Q, hTAPBPR^FLQ^ showed the tightest binding to MR1 C262S among all three mutants. In summary, our results demonstrate that TAPBPR residues contacting different MHC-I surfaces, including the α_2–1_ helix, are tolerant to optimization, by either introducing a minimal set of hTAPBPR substitutions or by selecting desired TAPBPR orthologs. Notably, TAPBPR provides a stable structural scaffold for obtaining gain-of-function interactions with allotypes that were previously not susceptible to interactions with the chaperone, such as HLA-A*01:01 and HLA-B*08:01.

## DISCUSSION

TAPBPR, a homolog of tapasin, is known for its dual roles as chaperone and peptide exchange catalyst with exquisite HLA allelic specificity (*32*). Although predicted structures of TAPBPR from different species are remarkably similar (*32*), whether different orthologs can mediate peptide exchange on human MHC-I proteins has not been addressed. Here, we demonstrated novel xeno interactions between a set of HLA allotypes and chTAPBPR distinct from the interaction profile with the human ortholog. Similar to hTAPBPR (*27*), chTAPBPR acts on properly conformed, peptide-loaded MHC-I molecules with a restricted allele selectivity (Figs. 2C and 3C and fig. S11A). We further investigated interactions between TAPBPR orthologs and MHC-I in its peptide-deficient form and demonstrated a broad allelic specificity and relatively high affinities toward empty molecules covering all six A supertypes, B08 and B44 supertypes, as well as HLA-E and HLA-G (Fig. 3G). We showed chTAPBPR-binding epitopes on empty, peptide-receptive HLA-B*08:01 located on the α_1_ and α_2–1_ helices of the heavy chain with a contribution from β_2_m (Fig. 5C), consistent with the footprint of hTAPBPR on murine MHC-I (*23*, *24*) and the conserved allosteric site underneath the α_2–1_ helix of MHC-I that is crucial for tapasin interactions (*12*). The interactions with peptide-deficient MHC-I molecules have been shown to provide an essential protein homeostasis mechanism by stabilizing misfolded, nascent MHC species inside the cell (*12*, *56*). Together, our data support two important hallmarks that (i) TAPBPR interactions are both MHC allele and peptide dependent with a broader allelic recognition of the empty molecules and (ii) TAPBPR can stabilize empty molecules and maintain them in a peptide-receptive state to facilitate exchange.

**Fig. 8.**
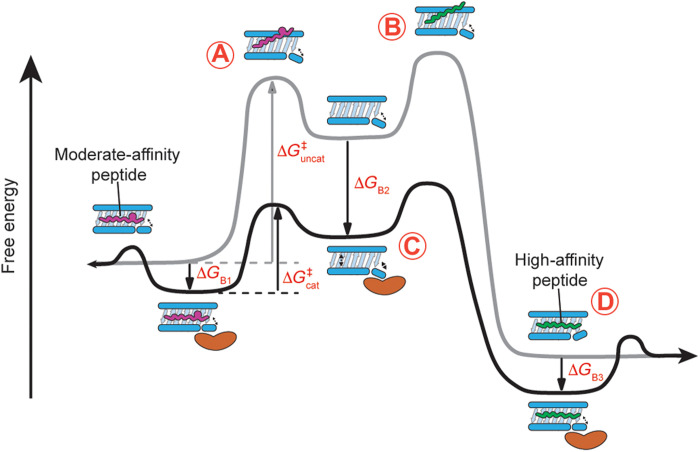
TAPBPR orthologs can enact multiple mechanisms to enable ligand exchange on MHC-I molecules. (**A**) MHC-I loaded with a moderate affinity placeholder peptide can spontaneously but slowly generate empty molecules. (**B**) These empty molecules overcome a high-activation energy barrier (Δ*G*^‡^uncat) to be in the transient peptide-receptive state for peptide loading. (**C**) A catalytic amount of TAPBPR functions to lower the activation energy barrier (Δ*G*^‡^cat) by recognizing the empty molecules with high affinity (binding free energy Δ*G*_B2_) and stabilizing an open conformation for peptide loading. (**D**) Loading of high-affinity peptides induces a closed MHC-I groove conformation leading to TAPBPR dissociation due to the substantially reduced affinity of TAPBPR toward peptide-loaded molecules (indicated by binding free energies Δ*G*_B1_ and Δ*G*_B3_). The reaction coordinate diagram from left to right shows the exchange of a moderate-affinity peptide to a high-affinity peptide, which can happen in the opposite direction with a much higher activation energy barrier and thus slower kinetics.

Furthermore, our results underline a strong correlation between the recognition of empty MHC-I molecules by different TAPBPR variants and the rate of peptide exchange reactions. We find that substoichiometric amounts of TAPBPR can substantially accelerate peptide exchange kinetics through tight interactions with empty MHC-I molecules (Figs. 3G, and 4, D, F, and H). According to this model, MHC-I loaded with a moderate affinity, placeholder peptide can undergo a slow, spontaneous process of peptide dissociation to create empty molecules (Fig. 8A). Subsequent binding of incoming peptides requires empty MHC-I to transit from a “closed” to an open groove state (*25*, *63*), which involves a new activation energy barrier (Fig. 8B). TAPBPR stabilizes the empty MHC-I groove in an open conformation with high affinity and enhances the transition rate between conformational states, thereby lowering the overall free energy (Fig. 8C) (*63*–*65*). Loading of high-affinity peptides induces a closed MHC-I groove conformation leading to TAPBPR dissociation due to the substantially increased *K*_D_ toward peptide-loaded molecules (Fig. 8D) (*25*, *50*). Nevertheless, in contrast to the classical, naturally occurring enzymes, TAPBPR exhibits a relatively minor catalytic effect [10- to 10^2^-fold versus 10^6^ to 10^12^-fold faster than the rate of uncatalyzed reaction (*66*)]. One possible reason for the reduced catalytic efficiency of TAPBPR is its additional requirement to function as a molecular chaperone of nascent MHC-I molecules inside the cell. According to its chaperone function, TAPBPR must bind empty MHC-I molecules with high affinity (the substrate for peptide loading). This requirement may compromise its ability to stabilize and extend the “active” state of MHC-I for the loading reaction. While previous studies have shown that hTAPBPR can recognize peptide-loaded molecules to promote suboptimal peptide dissociation (*18*, *23*, *60*), this unloading activity typically requires micromolar-range concentrations of hTAPBPR (fig. S6 A and B) and is likely not the primary cellular function of hTAPBPR. TAPBPR orthologs in a molar excess substantially enhance the overall exchange rate (Fig. 4H and figs. S7 to S9) and complete bulk peptide exchange in a short period of incubation (fig. S10). This mechanism of recognizing peptide-loaded MHC-I and forming a peptide/MHC-I/TAPBPR intermediate for peptide unloading may also explain why engineered hTAPBPR variants show improved peptide exchange function (Fig. 6D, and fig. S16, A to D). hTAPBPR mutants at a low concentration rather than a conventional molar excess can now promote peptide unloading due to their reduced *K*_D_ (increased affinity) to the peptide-loaded MHC-I (Fig. 7, E and F, and fig. S17). TAPBPR orthologs and engineered variants have differential respective binding-free energy (Δ*G*_B1_, Δ*G*_B1_, and Δ*G*_B3_) to distinct HLA allotypes, resulting in various levels of activation free energy barrier and, therefore, different peptide exchange kinetics. Together, our results demonstrate the catalytic effect of TAPBPR to promote the unloading of the peptide-loaded MHC-I and to stabilize a reservoir of empty, peptide-receptive molecules to capture new incoming peptides (Fig. 8).

Since the discovery of molecular chaperones tapasin (*6*, *67*) and TAPBPR (*8*, *9*), efforts to identify HLA alleles that are susceptible to chaperone-assisted peptide exchange in vitro or on the cell surface have been carried out using a range of approaches (*11*, *12*, *18*, *22*, *68*). Because of its ability to function independently of the PLC, TAPBPR is best suited for peptide exchange applications. Previous applications include the loading of immunogenic peptides onto surface-expressed MHC-I molecules (*62*) and the generation of pMHC-I tetramer libraries in a high-throughput manner for T cell epitope selection and antigen-specific T cell monitoring (*47*). However, TAPBPR-mediated peptide exchange has only been demonstrated for a limited set of common HLA allotypes, mainly from the A02 and A24 supertypes, limiting a wide adoption of these technologies in biomedical applications (*11*, *18*, *22*, *47*). Here, we showed novel, optimized peptide exchange functions on HLA-A*01:01, HLA-A*30:01, HLA-A*29:02, HLA-A*03:01, HLA-A*11:01, HLA-A*24:02, HLA-B*08:01, and HLA-B*37:01 using chTAPBPR (Fig. 4H), as well as identified hTAPBPR mutants that markedly enhanced peptide exchange efficiency on HLA-A*02:01 and enabled peptide exchange on HLA-A*01:01 (Fig. 6, D and F). Deep scanning mutagenesis, based on an indirect cell assay for interactions between hTAPBPR and folded HLA-A*02:01 localized on the cell surface, demonstrated that hTAPBPR residues in contact with the α_2–1_ helix of MHC-I had a higher tolerance for mutations compared to other regions at the interaction surface (Fig. 6, A and B), possibly reflecting imperfect complementarity at the interface to support MHC-I polymorphisms. Although the highly polymorphic nature of MHC-I molecules challenges the engineering of “universal” chaperones, we demonstrated a relatively conserved TAPBPR-binding epitope on MHC-I (Fig. 7A) and the possibility of designing and engineering off-the-shelf TAPBPR variants with minimal adjustments to enable peptide exchange on HLA allotypes of choice. Our study further opens the use of TAPBPR orthologs to perform ligand exchange on oligomorphic MHC-Ib and MR1 molecules. TAPBPR orthologs can also be used in various cancer immunotherapeutic settings to narrow the peptide repertoire, thereby increasing neoepitope immunogenicity. In conclusion, the knowledge gained by our studies can guide the design of engineered TAPBPR variants with tailored HLA specificity and catalytic efficiency for peptide exchange applications both in vitro and in vivo.

## MATERIALS AND METHODS

### Phylogenetic analysis

TAPBPR sequence references used for the phylogenetic analysis are as follows: cat (*Felis catus*), XP_023112473.2; chicken (*G. gallus*), NP_001382952.1; chimpanzee (*Pan troglodytes*), XP_009422944.1; dog (*Canis lupus familiaris*), XP_005637308.1; dolphin (*Tursiops truncatus*), XP_033723028.1; frog (*Xenopus laevis*), XP_018080550.1; goat (*Capra hircus*), XP_017904083.1; horse (*Equus caballus*), XP_023498789.1; human (*H. sapiens*), Q9BX59; mouse (*M. musculus*), XP_030111162.1; opossum (*Monodelphis domestica*), XP_007485846.1; rabbit (*Oryctolagus cuniculus*), XP_017198892.1; salmon (*Salmo salar*), NP_001133983.1; shark (*Carcharodon carcharias*), XP_041036048.1; turtle (*Chelydra serpentina*), KAG6922351.1; zebrafish (*Danio rerio*), XP_001919985.2. All amino acid sequence alignments were performed using ClustalOmega (*69*) and processed using ESPript (*70*); the tree was inferred using best-fit models as calculated by MEGA7 (*71*) and bootstrapped using 100 replicates. The predicted complex structures were generated using RosettaCM (*72*).

### Peptides and ligands

All peptide sequences are given as standard single-letter codes. High-affinity epitopic peptides for different HLA allotypes were selected by NetMHCpan4.1 and purchased from GenScript, Piscataway, USA, at >90% purity. βF containing placeholder peptides were synthesized in-house on 2-chlorotrityl resin using a CEM Liberty Blue automated microwave peptide synthesizer from 9-fluorenyl methoxycarbonyl (Fmoc)–protected amino acids (including Fmoc-β-Phe-OH) using iterative cycles of *N*,*N*′-diisopropylcarbodiimide/ethyl cyanohydroxyiminoacetate–mediated coupling and piperidine-mediated deprotection, both under microwave irradiation. Peptides were deprotected and cleaved from resin by treatment with trifluoroacetic acid/water/triisoproylsilane/phenol (88:5:5:2) for 1 to 3 hours. Solvent was removed under a flow of nitrogen, and peptides were precipitated with ice-cold ether. Peptides were subsequently purified by reverse phase chromatography eluting with 5 to 95% acetonitrile in water containing 0.05% trifluoroacetic acid over a C18 column. Peaks containing peptides were identified by liquid chromatography (LC)–MS, pooled, and concentrated in vacuo to yield a colorless solid. Photolabile peptides were purchased from Biopeptek Inc., Malvern, USA, or synthesized in-house as above and using J as Fmoc-3-amino-3-(2-nitrophenyl)-propionic acid. Peptides were solubilized in distilled water and centrifuged at 14,000 rpm for 15 min. The concentration of each peptide solution was measured and calculated using the respective absorbance and extinction coefficient at 205-nm wavelength. MR1 C262S ligand AC-6-FP was purchased from Cayman Chemical no. 23303.

### Recombinant protein expression, refolding, and purification

Plasmid DNA encoding the BirA substrate peptide (BSP; LHHILDAQKMVWNHR)–tagged luminal domain of MHC-I heavy chains and hβ_2_m light chain were provided by the National Institutes of Health tetramer facility (Emory University) and transformed into *Escherichia coli* BL21 (DE3) cells (New England Biolabs). Proteins were expressed in the Luria-Broth medium, and inclusion bodies were collected and purified as previously described (*73*). For the generation of pMHC-I molecules, in vitro refolding was performed by slowly diluting a 200-mg mixture of BirA-tagged MHC-I and hβ_2_m at a 1:3 molar ratio over 24 hours in refolding buffer [0.4 M l-arginine-HCl, 100 mM tris (pH 8), 2 mM EDTA, 4.9 mM reduced l-glutathione, and 0.57 mM oxidized l-glutathione] containing 10 mg of the placeholder peptide. The mixture was protected from light when refolded with photolabile peptides. Refolding proceeded for 4 days, and proteins were purified by SEC using a HiLoad 16/600 Superdex 75-pg column at 1 ml/min with 150 mM NaCl and 20 mM tris buffer (pH 8.0).

The luminal domain of human WT and mutants, chicken, and moTAPBPR proteins lacking the non-disulfide–bonded free cysteines (C94S for all hTAPBPR variants, C94A for chicken, and C98A for mouse) tagged with BSP and 6-His was stably expressed in the *Drosophila melanogaster* S2 cell line (*11*). The cultures were induced with 1 mM CuSO_4_, and after 4 days, the supernatant was collected. The secreted TAPBPR molecules were purified using a high-density metal affinity agarose resin (ABT, Madrid). The eluted proteins were further purified by SEC using a HiLoad 16/600 Superdex 200-pg column at a flow rate of 1 ml/min in 150 mM NaCl and 20 mM sodium phosphate buffer (pH 7.4).

### Purification of peptide-receptive MHC-I/TAPBPR

chTAPBPR was mixed with HLA-B*08:01/FLRGRAJGL at a 1:1.5 molar ratio. The mixture was incubated at room temperature (RT) for 1 hour, followed by 40-min UV irradiation at 365 nm and 30-min additional RT incubation. The complex was purified by SEC using a Superdex 200-pg Increase 10/300 GL, and the eluted peaks were further analyzed by SDS–polyacrylamide gel electrophoresis (PAGE) to identify all components.

### Native gel shift assay

Purified MHC-I molecules refolded with photo-labeled peptides (pMHC-I) and TAPBPR proteins were loaded into polyacrylamide gels alone at a concentration of 7 μM and as mixtures. For the mixtures, a 1:1 molar ratio of pMHC-I and TAPBPR samples at 7 μM each was prepared and incubated at RT for 1 hour. The samples were then UV-irradiated for another hour at 365 nm and were run at 90 V for 5.5 hours on a 12% polyacrylamide gel. Staining was performed using InstantBlue (Novus Biologicals).

### Biotinylation and tetramer formation

BSP-tagged TAPBPR proteins were biotinylated using the BirA biotin-protein ligase bulk reaction kit (Avidity), according to the manufacturer’s instructions. Biotinylated molecules were washed using Amicon Ultra centrifugal filter units with a 10-kDa membrane cutoff, and the level of biotinylation was evaluated by SDS-PAGE gel shift assay in the presence of excess streptavidin. Biotinylated TAPBPR was prepared at a final concentration of 2 mg/ml. Streptavidin–phycoerythrin (PE; Agilent Technologies Inc.) at 4:1 monomer/streptavidin molar ratio was added to TAPBPR over 10 time intervals every 10 min at RT in the dark. The resulting TAPBPR tetramers can be stored at 4°C for up to 4 weeks.

### SAB screen

TAPBPR (7 μM) in tetramers were mixed with 4 μl of the LABScreen SAB suspension (OneLambda Inc., CA, USA) in a 96-well plate. The samples were incubated for 1 hour and 550 rpm at RT, washed four times in wash buffer (OneLambda Inc., CA, USA) to remove excess tetramers, and resuspended in phosphate-buffered saline (PBS; pH 7.2). For the negative controls, beads were incubated with the anti–HLA class I antibody W6/32 (Abcam, ab22432) at 550 rpm for 30 min at RT and washed three times before tetramer addition. To test the levels of peptide-loaded MHC-I molecules on the beads, we used the same W6/32 antibody and the secondary anti-mouse PE-conjugated antibody (Abcam, ab97024) for detection. The levels of TAPBPR bound to the beads were measured using the Luminex 100 Liquid Array Analyzer System, and the results were analyzed in GraphPad Prism v9.

### Differential scanning fluorimetry

DSF was used to measure the thermal stability of the TAPBPR proteins and the pMHC-I molecules. TAPBPR protein (7 μM) or pMHC-I molecules with TAPBPR and the desired peptide at 1:7:70 TAPBPR/pMHC-I/peptide molar ratio were incubated for 2 hours at RT in the dark and then mixed with 10X SYPRO Orange dye in a buffer of 150 mM NaCl and 20 mM sodium phosphate (pH 7.2) to a final volume of 20 μl. Samples were loaded into MicroAmp Optical 384-well plate and ran in triplicates. The experiment was performed on a QuantStudio 5 real-time polymerase chain reaction (PCR) machine with excitation and emission wavelengths set to 470 and 569 nm. The thermal stabilities of pMHC complexes were compared by plotting the first derivative of each melting curve and extracting the peak as the melting temperature (*T*_m_). The thermal stability was measured by gradually increasing temperature at a rate of 1°C/min between 25° and 95°C. Data analysis and fitting were performed in GraphPad Prism v9.

### Surface plasmon resonance

SPR experiments were conducted in duplicate or triplicate using a BiaCore T200 instrument (Cytiva) in SPR buffer [150 mM NaCl, 20 mM sodium phosphate (pH 7.4), and 0.1% Tween 20]. Approximately 1000 resonance units of biotinylated hTAPBPR or chTAPBPR or hTAPBPR mutants were immobilized at 10 μl/min on a streptavidin-coated chip (GE Healthcare). Various concentrations (0, 0.05, 0.1, 0.25, 0.5, 0.75, 1, 2.5, 5, 7.5, 10, and 20 μM) of HLA molecules were selected and injected over the chip at 25°C at a flow rate of 30 μl/min for 60 s, followed by a buffer wash with 180-s dissociation time. For MR1 experiments, various concentrations (0, 5, 10, 15, 20, 25, 50, and 100 μM) were selected and used on hTAPBPR WT and mutants or chTAPBPR immobilized streptavidin chip. Before SPR experiments of UV-irradiated molecules, HLA molecules were diluted to the desired concentrations and UV-irradiated for 20 min at 365 nm. SPR data were then immediately acquired following a 20-min UV irradiation. The SPR sensorgrams, association/dissociation rate constants (*k*_a_ and *k*_d_), and equilibrium dissociation constants (*K*_D_) were analyzed using the surface-bound analysis settings in BiaCore T200 evaluation software (Cytiva) or fitted using one site-specific binding by GraphPad Prism v9. SPR sensorgrams and saturation curves were prepared in GraphPad Prism v9.

### Fluorescence polarization

Kinetic association of fluorescently labeled peptides and various pHLA-I was monitored by FP. pHLA-I was combined with graded concentrations of TAPBPR in FP buffer [150 mM NaCl, 20 mM sodium phosphate, and 0.05% Tween 20 (pH 7.4)] and the optimized concentration of fluorescently labeled peptide to achieve a polarization baseline between 0 and 50 mP, determined via serial dilution. _FITC_KSDPIVAQY (20 nM), _FITC_KTFPPTEPK (20 nM), _TAMRA_KLFGYPVYV (40 nM), _FITC_KLIETYFSK (20 nM), _TAMRA_KYNPIRTTF (6 nM), _FITC_KLRGRAYGL (20 nM), and _FITC_KEDLRVSSF (10 nM) were added to HLA-A*01:01, HLA-A*30:01, HLA-A*02:01 and HLA-A*68:02, HLA-A*03:01 and HLA-A*11:01, HLA-A*24:02, HLA-B*08:01, and HLA-B*37:01 in the presence of TAPBPR orthologs and mutants at various concentrations as indicated. The pMHC-I concentration remained constant across experiments at 200 nM. Fluorescently labeled peptides were directly added to the well plate to avoid extended incubation and loss of data. Wells were loaded with 100 μl of reaction, and triplicates for each condition were performed at RT. The kinetic association was monitored for 2 to 12 hours dependent on the kinetics, and polarization measurements were recorded every 60 to 130 s. Excitation and emission values used to monitor the fluorescence of tetramethylrhodamine (TAMRA)- and fluorescein isothiocyanate (FITC)–labeled peptides were 531 and 595 nm and 475 and 525 nm, respectively.

Raw parallel (*I*_II_) and perpendicular emission intensities (*I*_⊥_) were collected and converted to polarization (mP) values using the equation 1000 × {[*I*_II_ − (*G* × *I*_⊥_)] / [*I*_II_ + (*G* × *I*_⊥_)]}. G factors of 0.33 and 0.4 were optimized for TAMRA- and FITC-labeled peptides in calculating the overall FP. Data analysis method (*45*) was adapted, and data fitting was performed in GraphPad Prism v9.

### Isothermal titration calorimetry

ITC experiments between pHLA-B*08:01 and chTAPBPR constructs were obtained using a MicroCal VP-ITC system (Malvern Panalytical). All proteins were exhaustively dialyzed into the buffer [150 mM NaCl and 20 mM sodium phosphate (pH 7.2)] and filtered through a 0.22-μm polyethersulfone (PES) membrane. A syringe containing pHLA-B*08:01 at 150 μM with 0.45 mM peptide (FLRGRAJGL) was titrated into the calorimetry cell containing 5 μM TAPBPR and the same peptide (0.45 mM). Injection volumes of 10 μl were performed for a duration of 10 s and spaced 220 s apart to allow a complete return to baseline. Data were subtracted from a control, where a syringe containing pMHC-I at 150 μM with 0.45 mM FLRGRAJGL was titrated into the calorimetry cell containing buffer and the peptide (0.45 mM). Data were processed and analyzed with Origin software. Isotherms were fit using a one-site ITC binding model. The first data point was excluded from the analysis. Reported *K*_D_, −T*Δ*S*, and Δ*H* values were determined using the one-site binding model.

### Hydrogen/deuterium exchange mass spectrometry

HLA-B*08:01/FLRGRAJGL and purified HLA-B*08:01/chTAPBPR were dialyzed into equilibration buffer [150 mM NaCl and 20 mM sodium phosphate (pH 6.5) in H_2_O] and diluted to a stock concentration of 30 μM. HLA-B*08:01/FLRGRAJGL was then either (i) kept on ice without exposure to UV light or (ii) UV-exposed for 40 min at 4°C. Samples were prepared and injected manually for several deuterium-exchange incubation periods. HLA-B*08:01/FLRGRAJGL with or without UV irradiation and purified HLA-B*08:01/chTAPBPR (5 μl) were diluted with 20 μl of equilibration buffer (all H experiments, 0 s) or deuterium buffer [150 mM NaCl and 20 mM sodium phosphate (pD 6.5) in D_2_O] to 6 μM. The proteins were incubated with deuterium buffer for 20 s, 1 min, 3 min, and 10 min at RT and 15 min at 46°C for HLA-B*08:01/FLRGRAJGL or at 32°C for UV-irradiated HLA-B*08:01 and purified HLA-B*08:01/chTAPBPR as all the D samples to calculate ΔMass_100%_. The samples were then quenched with an equal volume of acidic buffer [150 mM NaCl, 1 M TCEP, and 20 mM sodium phosphate (pH 2.35) in H_2_O, 25 μl]. Quenched proteins (3 μM) were immediately injected for LC-MS/MS, in which integrated pepsin digestion was performed using the C8 5-μm Column and the Q Exactive Orbitrap Mass Spectrometer. Peptide fragments corresponding to HLA-B*08:01 (97% coverage) and hβ_2_m (94% coverage) were identified using Thermo Proteome Discoverer v2.4.

The percent deuterium uptake was back exchange–corrected for each time point using the following equation (*57*): ΔHDX= $\frac{\text{Δ}{\mathrm{Mass}}_{T}-\text{Δ}{\mathrm{Mass}}_{0\%}}{\text{Δ}{\mathrm{Mass}}_{100\%}-\text{Δ}{\mathrm{Mass}}_{0\%}}$. ExMs2 program was used to identify and analyze deuterated peptides. The kinetic plots and the scaled B factor for the structure plot were generated by Python 3 and PyMOL (*74*).

### Cell lines and expression plasmids for cell assays

Expi293F (Thermo Fisher Scientific) cells were cultured in Expi293 Expression media (Thermo Fisher Scientific) at 37°C, 125 rpm, and 8% CO_2_. The generation of tapasin-KO cells is previously described (*50*). HLA-A*01:01-knockin/tapasin-KO cells were generated by first transfecting Expi293F cells with EcoRV-linearized pCEP4-HLA-A*01:01 (untagged). The cells were selected with hygromycin B (100 μg/ml) and FACS-sorted for HLA-A*01:01–positive cells after staining with anti-HLA-A*01:01 clone 8.L.101 (USBio, H6098-05). Following sorting, the HLA-A*01:01–positive cells were transfected with plasmids encoding human codon–optimized Cas9 and tapasin guide RNA, as previously described (*50*) and then FACS-sorted for cells that no longer processed HLA-A*01:01 to the cell surface.

TAPBPR-TM was cloned into pCEP4 (Invitrogen) with a canonical N-terminal MHC-I signal peptide, FLAG tag, linker, luminal domain of hTAPBPR, linker, and TM domain of HLA-G (Addgene, no. 135500). Mouse and chTAPBPR were cloned using the same design and included C98A and C94A, respectively. HLA-A*01:01 was subcloned by removing the myc tag from pCEP4-myc- HLA-A*01:01, as previously described (*27*). Site-directed mutagenesis of TAPBPR-TM mutants was achieved using overlap extension PCR. The inserts of all plasmids were confirmed by Sanger sequencing.

### Deep mutational scan

A single SSM library was constructed for TAPBPR-TM, focused on specific residues in the luminal domain that interface with MHC-I or are at control sites. The SSM library was created by subcloning the previously published TAPBPR-tapasinCT libraries (*50*); a DNA fragment corresponding to the TAPBPR luminal domain was PCR-amplified and a canonical TM tail from HLA-G added in a second round of PCR-based assembly. The resulting TAPBPR-TM library products were pooled, restriction enzyme–digested, and ligated into pCEP4 before electroporation into NEB 10-beta cells (New England Biolabs). The transformation efficiency was at least 10-fold higher than the theoretical sequence diversity. Purified library plasmid DNA (1 ng/ml of culture) was diluted with pCEP4-ΔCMV (*75*) (1500 ng/ml of culture) and transfected using ExpiFectamine (Thermo Fisher Scientific) into tapasin-KO Expi293F cells (2 × 10^6^/ml). The medium was replaced after 2 hours. Under these conditions, cells typically receive no more than a single coding plasmid (*76*). Cells were collected 24 hours after transfection (400*g* for 3 min at 4°C), washed with PBS containing 0.2% bovine serum albumin (PBS-BSA), incubated for 30 min at 4°C with 1:200 anti–HLA-A*02:01 PE (BioLegend, clone BB7.2), washed twice with PBS-BSA, and sorted on a BD FACSAria II. 4′,6-Diamidino-2-phenylindole (300 nM final concentration) was added, and cells were gated for viability. The main singlet population was then gated by scattering, and the top 2% of cells for PE fluorescence were collected (fig. S15). RNA was extracted (GeneJet RNA purification kit, Thermo Fisher Scientific), and first-strand cDNA was synthesized using Accuscript (Agilent) primed with EBV reverse primer, which anneals to the 3′ untranslated region of pCEP4-encoded transcripts. PCR products covering the mutated regions in the TAPBPR-TM library were amplified, followed by a second round of PCR to add Illumina adapters and sample-specific barcodes. The amplicons were Illumina-sequenced on a NovaSeq 6000 and analyzed with Enrich (*77*). Briefly, the frequency of each sequence variant in the sorted library was divided by its frequency in the presorted naive library to calculate an enrichment ratio. The enrichment ratios are presented on a log_2_ scale. Conservation scores from the sorting experiment were calculated by averaging the log_2_ enrichment ratios for all amino acid substitutions at a given residue (excluding nonsense mutations).

### Flow cytometry

Expi293F cells were transfected at 2 × 10^6^ cells/ml using ExpiFectamine (Thermo Fisher Scientific) and collected 24 hours after transfection. For testing surface HLA-A*02:01, cells were washed with PBS-BSA, incubated with 1:200 anti–HLA-A*02:01 PE (BioLegend, clone BB7.2, 343306) and 1:200 anti-DYKDDDK APC (BioLegend, clone L5, 637308) for staining surface TAPBPR, washed twice with PBS-BSA, and analyzed on a BD Accuri cytometer. For measuring surface HLA-A*01:01, cells were washed with PBS-BSA, incubated with 1:200 mouse anti–HLA-A*01:01 (USBio, clone 8.L.101, H6098-05), washed twice with PBS-BSA, incubated with 1:200 goat anti-mouse IgG + IgM (H + L) (Jackson ImmunoResearch), washed twice with PBS-BSA, and analyzed on a BD Accuri. Results were processed using FCS Express 6.

### Immunoblots

Equal cell quantities were lysed with SDS-loading dye. Proteins were separated by gel electrophoresis and transferred to a polyvinylidene difluoride (PVDF) membrane. PVDF membranes for FLAG-tagged TAPBPR proteins were blocked with 3% BSA in tris-buffered saline supplemented with 0.2% Tween 20 (TBS-T) for 30 min, incubated with 1:2000 anti-FLAG alkaline phosphatase (Sigma-Aldrich) in 1% BSA/TBS-T, washed five times with TBS-T, and then visualized with one-step bromochloroindolyl phosphate (Thermo Fisher Scientific). Cyclophilin B (PP1B) was used as a loading control; blots were blocked with 5% nonfat milk/TBS-T, incubated with 1:2000 rabbit anti-cyclophilin B (Invitrogen), washed five times with TBS-T, incubated with 1:10,000 goat anti-rabbit horseradish peroxidase (Jackson ImmunoResearch), washed five times with TBS-T, and visualized using Clarity Western ECL Substrate (Bio-Rad).

## Notes

View/request a protocol for this paper from *Bio-protocol*.
