# Supplementary material for: Xeno interactions between MHC-I proteins and molecular chaperones enable ligand exchange on a broad repertoire of HLA allotypes
Source: Sci Adv. 2023 Feb 24;9(8):eade7151. doi: 10.1126/sciadv.ade7151 (PMC9956121; doi:10.1126/sciadv.ade7151)
Supplement: Supplementary file 1 — Figs. S1 to S17 Tables S1 to S4 [file sciadv.ade7151_sm.pdf]

Science Advances
AAAS

Supplementary Materials for

Xeno interactions between MHC-I proteins and molecular chaperones enable ligand exchange on a broad repertoire of HLA allotypes

Yi Sun *et al.*

Corresponding author: Nikolaos G. Sgourakis, nikolaos.sgourakis@pennmedicine.upenn.edu

Sci. Adv. **9**, eade7151 (2023)
DOI: 10.1126/sciadv.ade7151

This PDF file includes:

Figs. S1 to S17
Tables S1 to S4

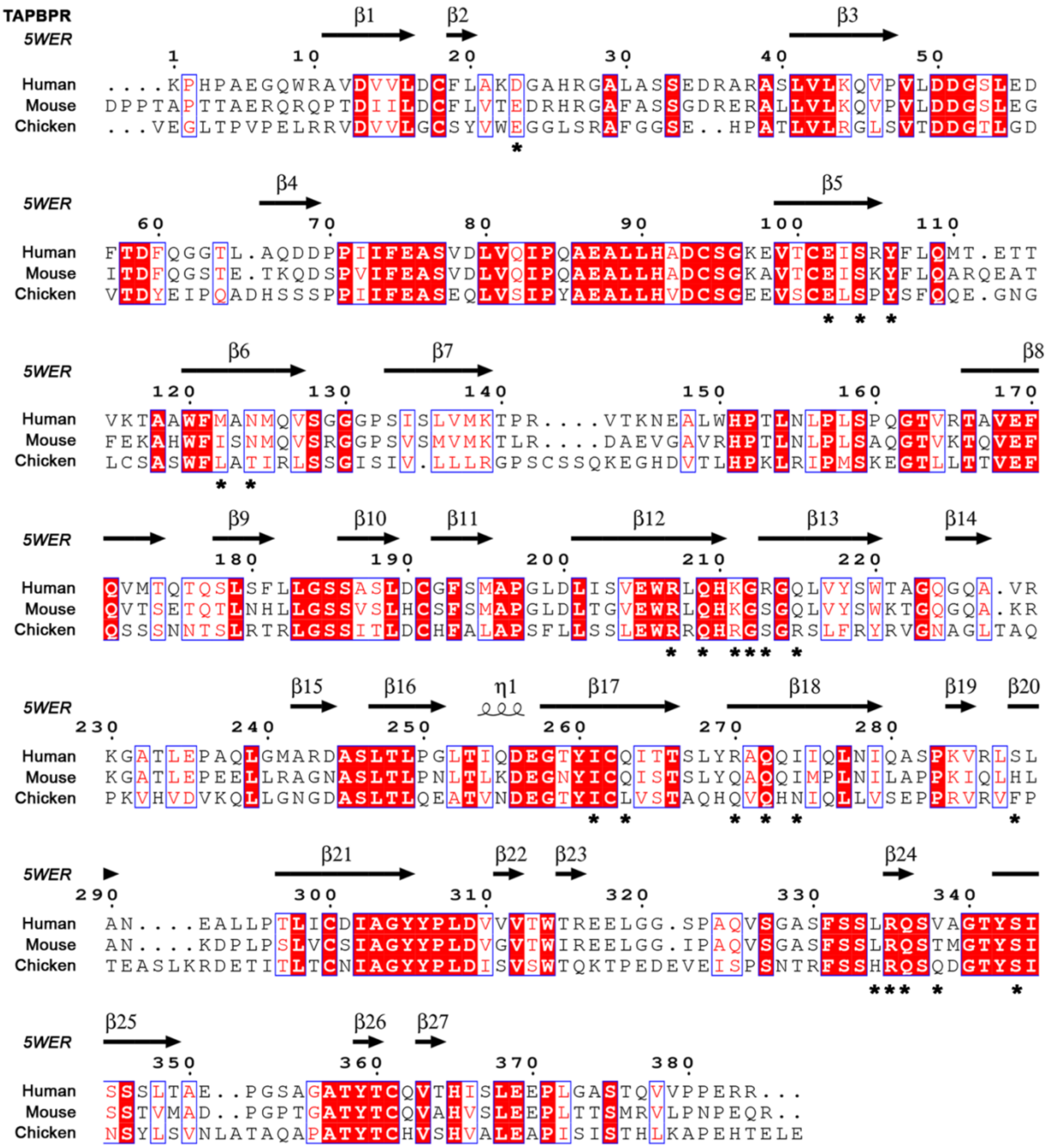

Figure S1. Sequence alignment between human, mouse, and chicken TAPBPR. Alignment of the luminal domains of human (UniProtKB/Swiss-Prot: Q9BX59), mouse (XP_030111162.1), and chicken (NP_001382952.1) TAPBPR. Conserved residues are marked in blue boxes, and the residues that are in direct contact with the MHC-I heavy chain based on the X-ray structure of the

H2-D^d/hTAPBPR complex[1] are noted with asterisks (*). The secondary structure of human TAPBPR (PDB ID: 5WER) is provided as a reference.

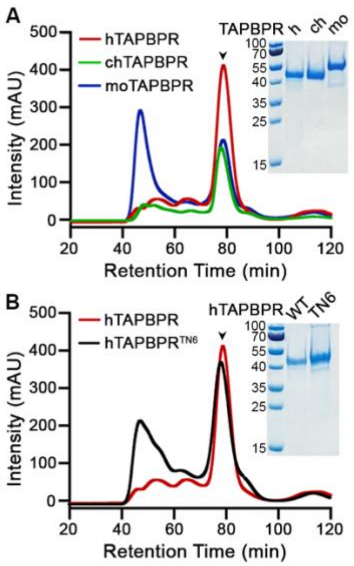

Figure S2. Purification of recombinant TAPBPR proteins. (A)-(B), Size exclusion chromatography (SEC) traces of **(A)** hTAPBPR, chTAPBPR, moTAPBPR, and **(B)** hTAPBPR^{TN6} proteins. The protein peaks are indicated by the arrow and further confirmed by SDS/PAGE analysis.

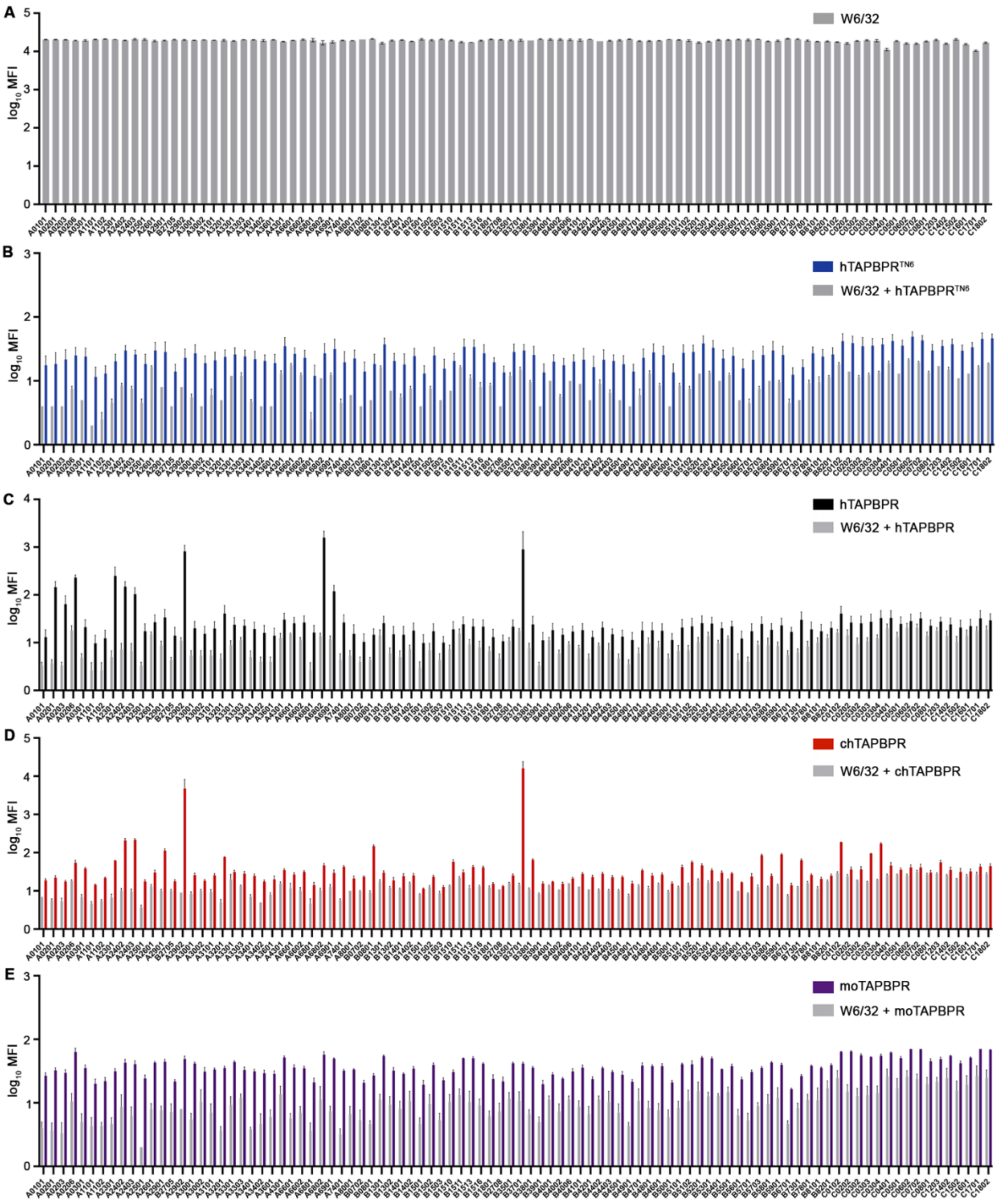

Figure S3. Binding levels of TAPBPR tetramers on HLA single antigen beads. (A), Levels of folded MHC-I molecules captured on single antigen beads (SABs), related to Figure 2, were detected using the primary anti-HLA Class I antibody W6/32 (Abcam, ab22432) and the secondary

anti-mouse PE-conjugated antibody (Abcam, ab97024). Similar levels of peptide-loaded MHC-I molecules were observed across different HLA allotypes. **(B)-(E),** Bar graphs showing the logarithm of Mean Fluorescent Intensity (MFI) levels of tetramerized **(B)** $hTAPBPR^{TN6}$ (negative control), **(C)** hTAPBPR, **(D)** chTAPBPR, and **(E)** moTAPBPR binding to HLA molecules on SABs. Incubation with the W6/32 antibody prior to tetramer addition was used to control for background staining levels. The plotted data were generated based on n=3 independent experiments and the standard deviation is depicted with error bars.

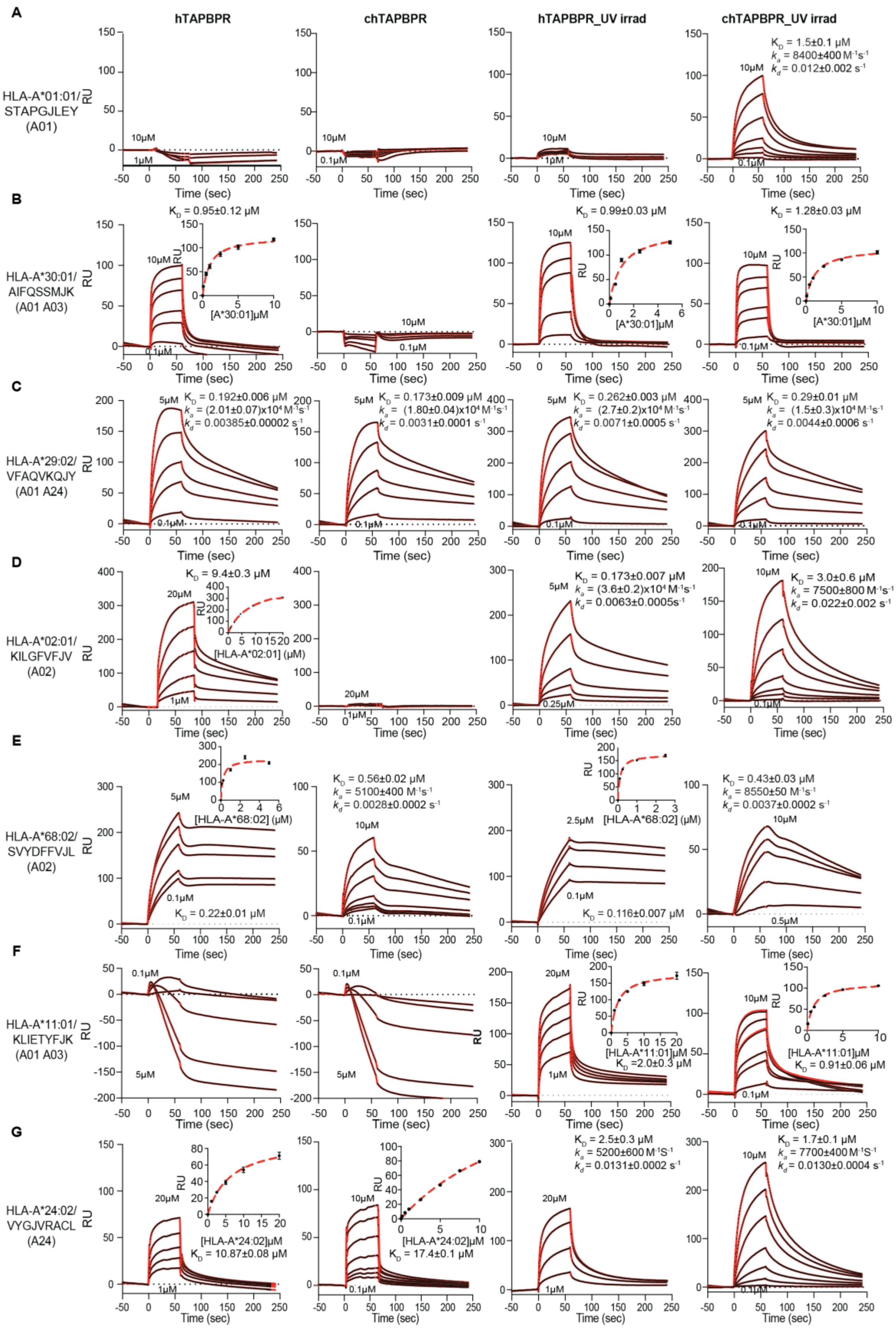

A
hTAPBPR
chTAPBPR
hTAPBPR_UV irrad
chTAPBPR_UV irrad
HLA-A*01:01/
STAPGJLEY
(A01)
K_D = 1.5±0.1 μM
k_a = 8400±400 M⁻¹s⁻¹
k_d = 0.012±0.002 s⁻¹
B
HLA-A*30:01/
AIFQSSMJK
(A01 A03)
K_D = 0.95±0.12 μM
K_D = 0.99±0.03 μM
K_D = 1.28±0.03 μM
[A*30:01]μM
C
HLA-A*29:02/
VFAQVKQJY
(A01 A24)
K_D = 0.192±0.006 μM
k_a = (2.01±0.07)x10⁴ M⁻¹s⁻¹
k_d = 0.00385±0.00002 s⁻¹
K_D = 0.173±0.009 μM
k_a = (1.80±0.04)x10⁴ M⁻¹s⁻¹
k_d = 0.0031±0.0001 s⁻¹
K_D = 0.262±0.003 μM
k_a = (2.7±0.2)x10⁴ M⁻¹s⁻¹
k_d = 0.0071±0.0005 s⁻¹
K_D = 0.29±0.01 μM
k_a = (1.5±0.3)x10⁴ M⁻¹s⁻¹
k_d = 0.0044±0.0006 s⁻¹
D
HLA-A*02:01/
KILGFVFJV
(A02)
K_D = 9.4±0.3 μM
[HLA-A*02:01] (μM)
K_D = 0.173±0.007 μM
k_a = (3.6±0.2)x10⁴ M⁻¹s⁻¹
k_d = 0.0063±0.0005s⁻¹
K_D = 3.0±0.6 μM
k_a = 7500±800 M⁻¹s⁻¹
k_d = 0.022±0.002 s⁻¹
E
HLA-A*68:02/
SVYDFFVJL
(A02)
[HLA-A*68:02] (μM)
K_D = 0.22±0.01 μM
K_D = 0.56±0.02 μM
k_a = 5100±400 M⁻¹s⁻¹
k_d = 0.0028±0.0002 s⁻¹
K_D = 0.116±0.007 μM
K_D = 0.43±0.03 μM
k_a = 8550±50 M⁻¹s⁻¹
k_d = 0.0037±0.0002 s⁻¹
F
HLA-A*11:01/
KLIETYFJK
(A01 A03)
[HLA-A*11:01]μM
K_D = 2.0±0.3 μM
K_D = 0.91±0.06 μM
G
HLA-A*24:02/
VYGJVRACL
(A24)
[HLA-A*24:02]μM
K_D = 10.87±0.08 μM
K_D = 17.4±0.1 μM
K_D = 2.5±0.3 μM
k_a = 5200±600 M⁻¹S⁻¹
k_d = 0.0131±0.0002 s⁻¹
K_D = 1.7±0.1 μM
k_a = 7700±400 M⁻¹S⁻¹
k_d = 0.0130±0.0004 s⁻¹
RU
Time (sec)

Figure S4. Direct interactions between HLA-A allotypes and TAPBPR orthologs. (A)-(G), SPR sensorgrams of various concentrations of peptide-loaded or -deficient **(A)** HLA-A*01:01, **(B)** HLA-A*30:01, **(C)** HLA-A*29:02, **(D)** HLA-A*02:01, **(E)** HLA-A*68:02, **(F)** HLA-A*11:01, and **(G)** HLA-A*24:02 flowed over a streptavidin chip coupled with hTAPBPR or chTAPBPR-biotin. The concentrations of analyte for the top and the bottom sensorgrams are noted. Data are mean ± σ, where n=2 for HLA-A*01:01, HLA-A*02:01 (chTAPBPR), and HLA-A*24:02 (chTAPBPR), and n=3 for HLA-A*30:01, HLA-A*29:02, HLA-A*02:01 (hTAPBPR), HLA-A*68:02, HLA-A*11:01, and HLA-A*24:02 (hTAPBPR). K_D, equilibrium constant; k_a, association rate constant; k_d, dissociation rate constant; RU, resonance units.

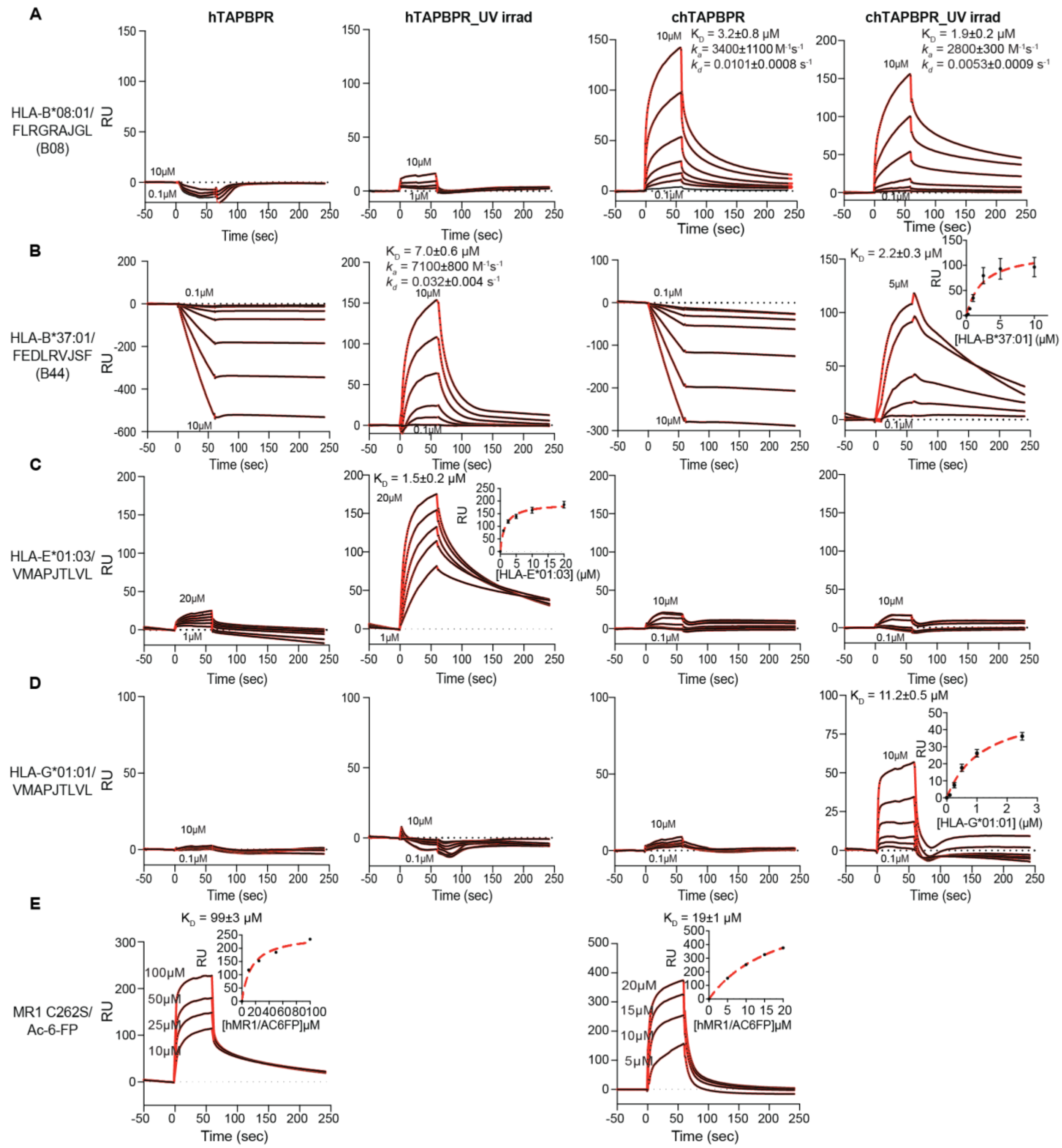

Figure S5. Direct interactions between HLA-B, MHC-Ib, or MR1 molecules and TAPBPR orthologs. (A)-(D), SPR sensorgrams of various concentrations of peptide-loaded or -deficient **(A)** HLA-B*08:01, **(B)** HLA-B*37:01, **(C)** HLA-E*01:03, and **(D)** HLA-G*01:01 flowed over a streptavidin chip coupled with hTAPBPR or chTAPBPR-biotin. **(E),** SPR sensorgrams of various concentrations of Ac-6-FP loaded MR1 C262S flowed over a streptavidin chip coupled with

hTAPBPR or chTAPBPR-biotin. The concentrations of analyte for the top and the bottom sensorgrams are noted. Data are mean ± σ, where n=2 for UV irradiated HLA-B*08:01, HLA-B*37:01 (hTAPBPR), HLA-E*03:01, and HLA-G*01:01, and n=3 for HLA-B*08:01, HLA-B*37:01 (chTAPBPR), and MR1. K_D, equilibrium constant; k_a, association rate constant; k_d, dissociation rate constant; RU, resonance units.

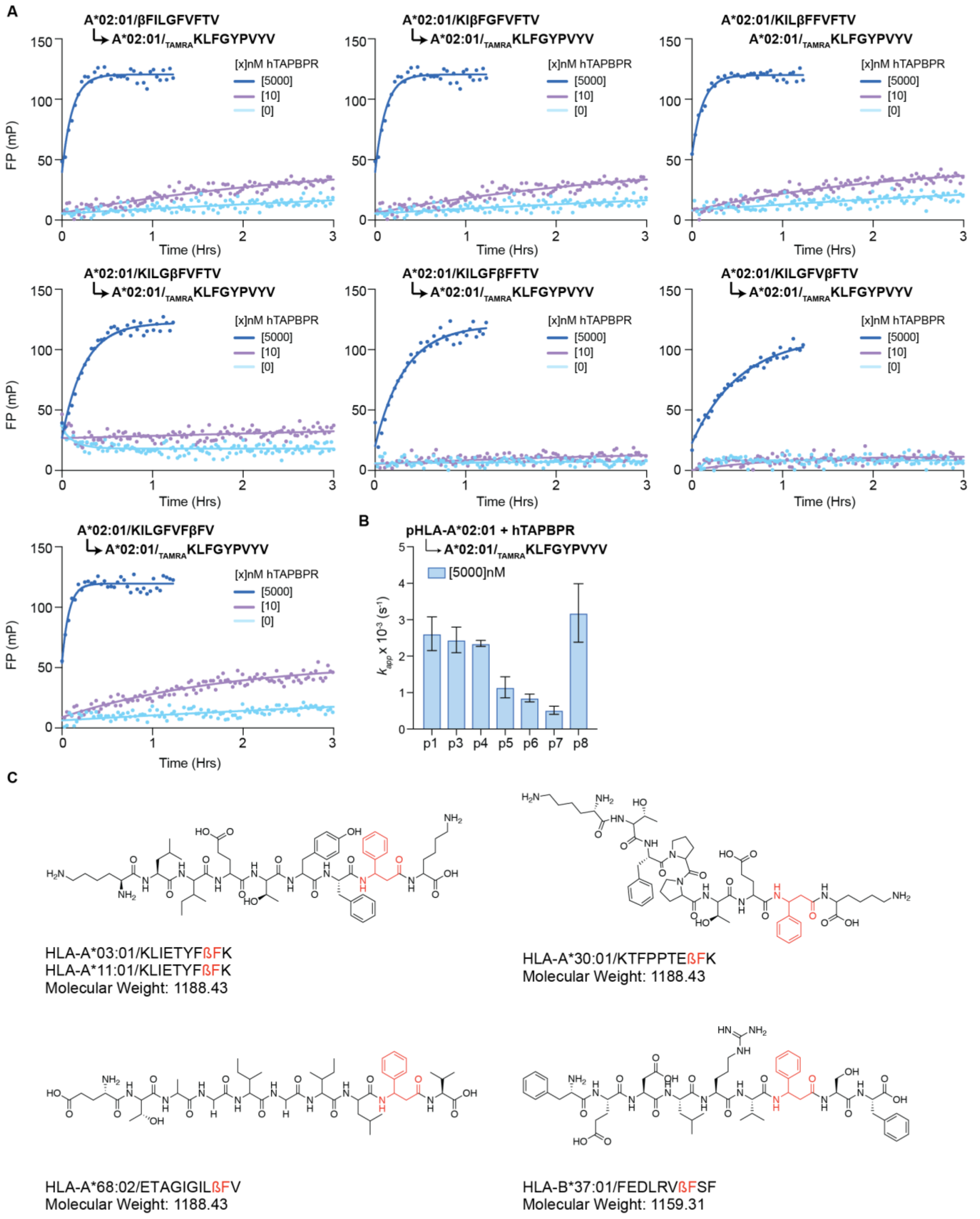

Figure S6. Structure-guided design of conditional peptide ligands influences TAPBPR-mediated peptide exchange kinetics for different HLA allotypes. (A), Assessing the effect of

the L-β-Phenylalanine residue (βF) at different peptide positions of HLA-A*02:01-restricted KILGFVFTV by fluorescence polarization (FP). The association profiles of the fluorophore-conjugated peptide $_{\text{TAMRA}}$KLFGYPVYV to HLA-A*02:01/βFILGFVFTV, KIβFGFVFTV, KILβFFVFTV, KILGβFVFTV, KILGFβFFTV, KILGFVβFTV, KILGFVFβFV without hTAPBPR, with 10 nM or 5000 nM hTAPBPR, as indicated. The data were fitted to a monoexponential association model to determine the apparent rate constant k_{app}. Results of three replicates (mean) are plotted. **(B),** The comparison of k_{app} for fluorescent peptide binding to HLA-A*02:01/KILGFVFTV with βF substitution at indicated positions in the presence of 5000 nM hTAPBPR. The apparent rate constant k_{app} was determined by fitting the raw trace to a monoexponential association model. Results of three replicates (mean ± σ) are plotted. **(C),** Structure-guided design of conditional peptide ligands for different HLA allotypes. The βF peptide ligands KLIETYFβFK, KTFPPTEβFK, ETAGIGILβFV, and FEDLRVβFSF are based on the HLA-A*03:01 immunodominant proteolipid protein (PLP) epitope KLIETYFSK, HLA-A*30:01 SARS-CoV-2 nucleoprotein epitope KTFPPTEPK, HLA-A*68:02 peptide ETAGIGILTV, and HLA-B*37:01 Influenza NP338-346 peptide FEDLRVLSF.

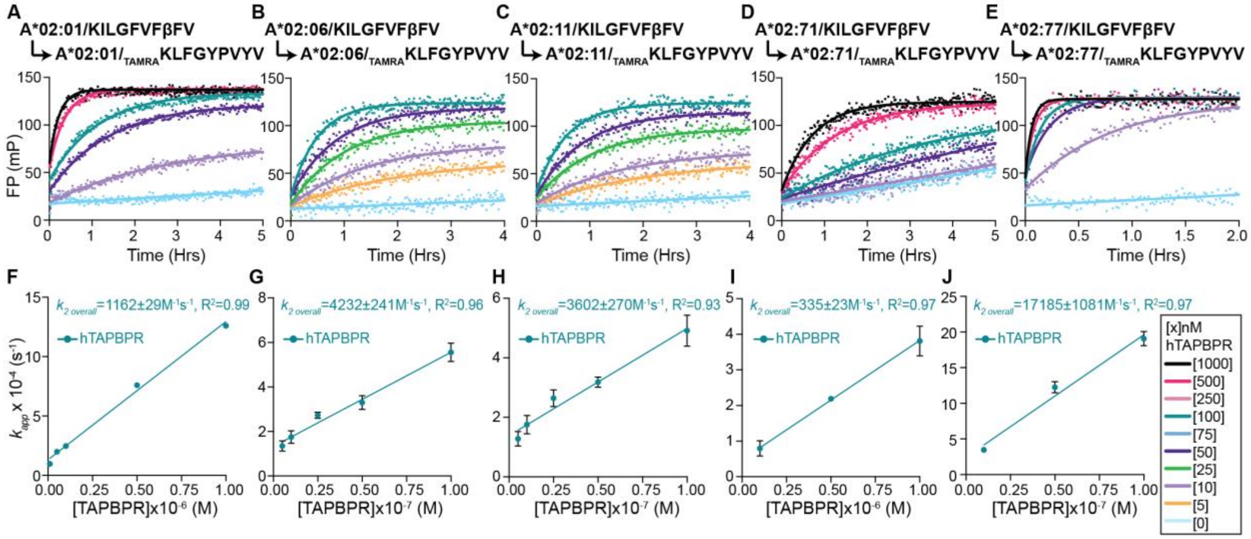

Figure S7. Effect of HLA-A*02 micropolymorphisms on peptide exchange by hTAPBPR. (A)-(E), Association profiles of the fluorophore-conjugated peptide $_{TAMRA}$KLFGYPVYV to KILGFVFβFV loaded **(A)** HLA-A*02:01, **(B)** HLA-A*02:06, **(C)** HLA-A*02:11, **(D)** HLA-A*02:71, and **(E)** HLA-A*02:77 in the presence of hTAPBPR at various concentrations, as indicated. The data were fitted to a monoexponential association model to determine apparent rate constants k_{app}. Results of three replicates (mean) are plotted. **(F)-(J),** Linear correlations between the apparent rate constants k_{app} and the concentrations of hTAPBPR for **(F)** HLA-A*02:01, **(G)** HLA-A*02:06, **(H)** HLA-A*02:11, **(I)** HLA-A*02:71, and **(J)** HLA-A*02:77. The extrapolation of the slope determines the overall rate $k_{2overall}$. Results of three replicates (mean ± σ) are plotted.

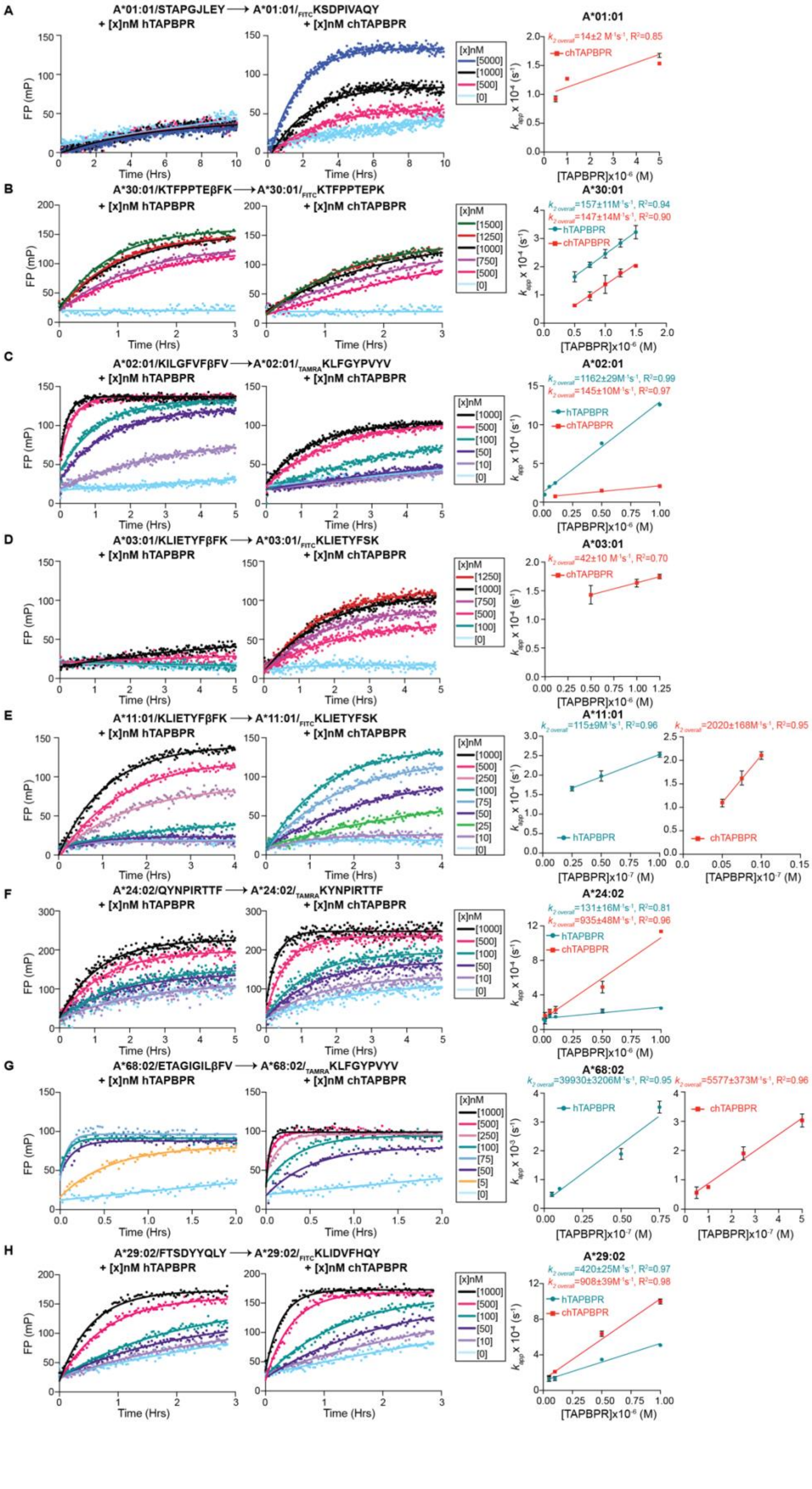
A
A*01:01/STAPGJLEY → A*01:01/FITCKSDPIVAQY
+ [x]nM hTAPBPR
+ [x]nM chTAPBPR
FP (mP)
Time (Hrs)
[x]nM
[5000]
[1000]
[500]
[0]
A*01:01
k2 overall=14±2 M-1s-1, R2=0.85
chTAPBPR
kapp x 10-4 (s-1)
[TAPBPR]x10-6 (M)
B
A*30:01/KTFPPTEβFK → A*30:01/FITCKTFPPTEPK
+ [x]nM hTAPBPR
+ [x]nM chTAPBPR
[1500]
[1250]
[1000]
[750]
[500]
[0]
A*30:01
k2 overall=157±11M-1s-1, R2=0.94
k2 overall=147±14M-1s-1, R2=0.90
hTAPBPR
chTAPBPR
C
A*02:01/KILGFVFβFV → A*02:01/TAMRAKLFGYPVYV
+ [x]nM hTAPBPR
+ [x]nM chTAPBPR
[1000]
[500]
[100]
[50]
[10]
[0]
A*02:01
k2 overall=1162±29M-1s-1, R2=0.99
k2 overall=145±10M-1s-1, R2=0.97
hTAPBPR
chTAPBPR
D
A*03:01/KLIETYFβFK → A*03:01/FITCKLIETYFSK
+ [x]nM hTAPBPR
+ [x]nM chTAPBPR
[1250]
[1000]
[750]
[500]
[100]
[0]
A*03:01
k2 overall=42±10 M-1s-1, R2=0.70
chTAPBPR
E
A*11:01/KLIETYFβFK → A*11:01/FITCKLIETYFSK
+ [x]nM hTAPBPR
+ [x]nM chTAPBPR
[1000]
[500]
[250]
[100]
[75]
[50]
[25]
[10]
[0]
A*11:01
k2 overall=115±9M-1s-1, R2=0.96
k2 overall=2020±168M-1s-1, R2=0.95
hTAPBPR
chTAPBPR
[TAPBPR]x10-7 (M)
F
A*24:02/QYNPIRTTF → A*24:02/TAMRAKYNPIRTTF
+ [x]nM hTAPBPR
+ [x]nM chTAPBPR
[1000]
[500]
[100]
[50]
[10]
[0]
A*24:02
k2 overall=131±16M-1s-1, R2=0.81
k2 overall=935±48M-1s-1, R2=0.96
hTAPBPR
chTAPBPR
G
A*68:02/ETAGIGILβFV → A*68:02/TAMRAKLFGYPVYV
+ [x]nM hTAPBPR
+ [x]nM chTAPBPR
[1000]
[500]
[250]
[100]
[75]
[50]
[5]
[0]
A*68:02
k2 overall=39930±3206M-1s-1, R2=0.95
k2 overall=5577±373M-1s-1, R2=0.96
hTAPBPR
chTAPBPR
kapp x 10-3 (s-1)
H
A*29:02/FTSDYYQLY → A*29:02/FITCKLIDVFHQY
+ [x]nM hTAPBPR
+ [x]nM chTAPBPR
[1000]
[500]
[100]
[50]
[10]
[0]
A*29:02
k2 overall=420±25M-1s-1, R2=0.97
k2 overall=908±39M-1s-1, R2=0.98
hTAPBPR
chTAPBPR

Figure S8. HLA-A peptide exchange kinetics by human vs. chicken TAPBPR. (A)-(G), The association profiles of fluorophore-conjugated peptide $_{FITC}$KSDPIVAQY, $_{FITC}$KTFPPTEPK, $_{TAMRA}$KLFGYPVYV, $_{FITC}$KLIETYFSK, $_{TAMRA}$KYNPIRTTF, and $_{FITC}$KLIDVFHQY to **(A)** HLA-A*01:01/STAPGJLEY, **(B)** HLA-A*30:01/KTFPPTEβFK, **(C)** HLA-A*02:01/KILGFVFβFV, **(D)** HLA-A*03:01/KLIETYFβFK, **(E)** HLA-A*11:01/KLIETYFβFK, **(F)** HLA-A*24:02/QYNPIRTTF, **(G)** HLA-A*68:02/ETAGIGILβFV, and **(H)** HLA-A*29:02/FTSDYYQLY in the presence of human or chicken TAPBPR at various concentrations, as indicated. Results of three replicates (mean) are plotted. The data were fitted to a monoexponential association model to determine apparent rate constants k_{app}. Linear correlations between the apparent rate constants k_{app} and the concentrations of TAPBPR orthologs were determined. The extrapolation of the slope determines the overall rate $k_{2overall}$. Results of three replicates (mean ± σ) are plotted.

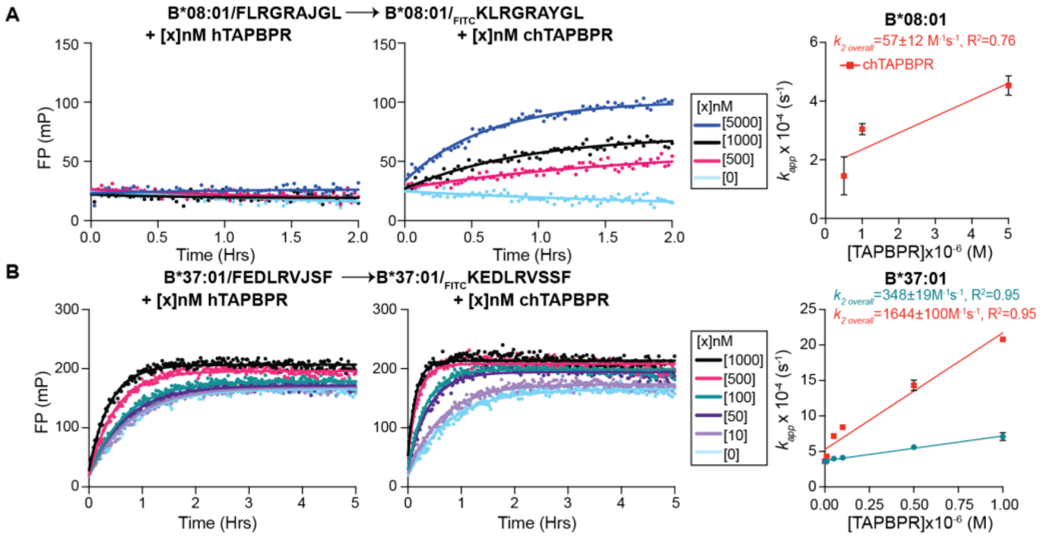

Figure S9. HLA-B peptide exchange kinetics by human vs. chicken TAPBPR. (A)-(B), The association profiles of fluorophore-conjugated peptide $_{FITC}$KLRGRAYGL and $_{FITC}$KEDLRVSSF to **(A)** HLA-B*08:01/FLRGRAJGL and **(B)** HLA-B*37:01/FEDLRVJSF in the presence of human or chicken TAPBPR at various concentrations, as indicated. Results of three replicates (mean) are plotted. The data were fitted to a monoexponential association model to determine apparent rate constants k_{app}. Linear correlations between the apparent rate constants k_{app} and the concentrations of TAPBPR orthologs were determined. The extrapolation of the slope determines the overall rate $k_{2overall}$. Results of three replicates (mean ± σ) are plotted.

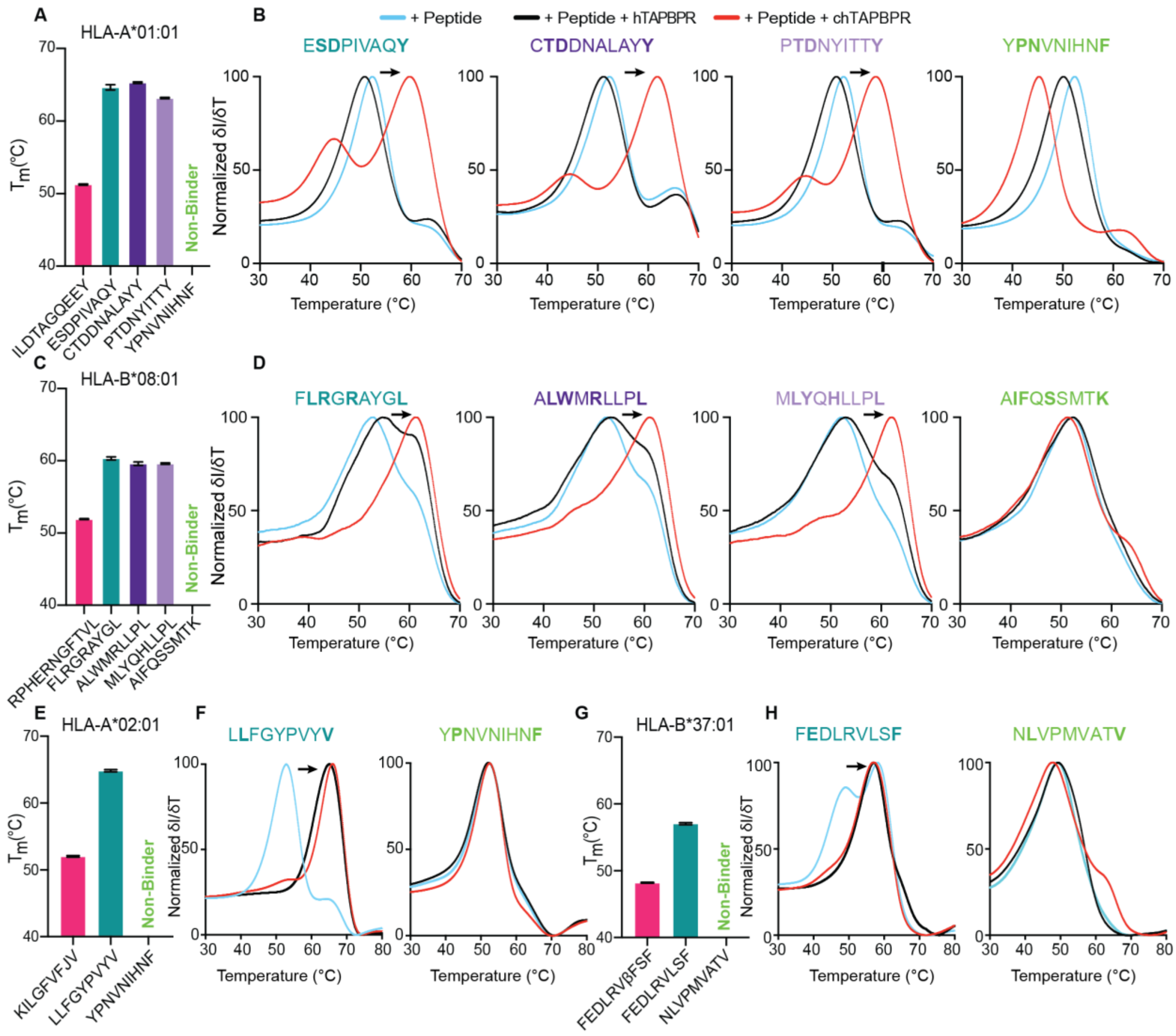

Figure S10. TAPBPR orthologs catalyze a complete exchange of HLA-A*01:01, B*08:01, A*02:01, and B*37:01 for high-affinity peptides. (A), Melting temperature (T_m, °C) obtained from differential scanning fluorimetry (DSF) of HLA-A*01:01 bound to ILDTAGQEEY, ESDPIVAQY, CTDDNALAYY, or PTDNYITTY. Data are mean ± σ for n = 3 technical replicates. **(B),** Thermal stabilities of HLA-A*01:01/ILDTAGQEEY were determined in the presence of 10-fold molar excess ESDPIVAQY, CTDDNALAYY, PTDNYITTY, or a non-specific peptide YPNVNIHNF for two-hour incubation at room temperature (RT) (light blue) with a catalytic amount of human (black) or chicken (red) TAPBPR. **(C),** Melting temperature (T_m, °C)

obtained from DSF of HLA-B*08:01 bound to peptide RPHERNGFTVL, FLRGRAYGL, ALWMRLLPL, or MLYQHLLPL. Data are mean ± σ for n = 3 technical replicates. **(D),** Thermal stabilities of HLA-B*08:01/RPHERNGFTVL were determined in the presence of 10-fold molar excess FLRGRAYGL, ALWMRLLPL, MLYQHLLPL, or a non-specific peptide AIFQSSMTK for two-hour incubation at RT (light blue) with a catalytic amount of human (black) or chicken (red) TAPBPR. **(E),** Melting temperature (T_m, °C) obtained from DSF of HLA-A*02:01 bound to KILGFVFJV and LLFGYPVYV. Data are mean ± σ for n = 3 technical replicates. **(F),** Thermal stabilities of HLA-A*02:01/KILGFVFJV in the presence of 10-fold molar excess LLFGYPVYV and a non-specific peptide YPNVNIHNF for two-hour incubation at RT (light blue) with a catalytic amount of human (black) or chicken (red) TAPBPR. **(G),** Melting temperature (T_m, °C) obtained from DSF of HLA-B*37:01 bound to FEDLRVβFSF and FEDLRVLSF. Data are mean ± σ for n = 3 technical replicates. **(H),** Thermal stabilities of HLA-B*37:01/FEDLRVβFSF in the presence of 10-fold molar excess FEDLRVLSF and a non-specific peptide NLVPMVATV for two-hour incubation at RT (light blue) with a catalytic amount of human (black) or chicken (red) TAPBPR.

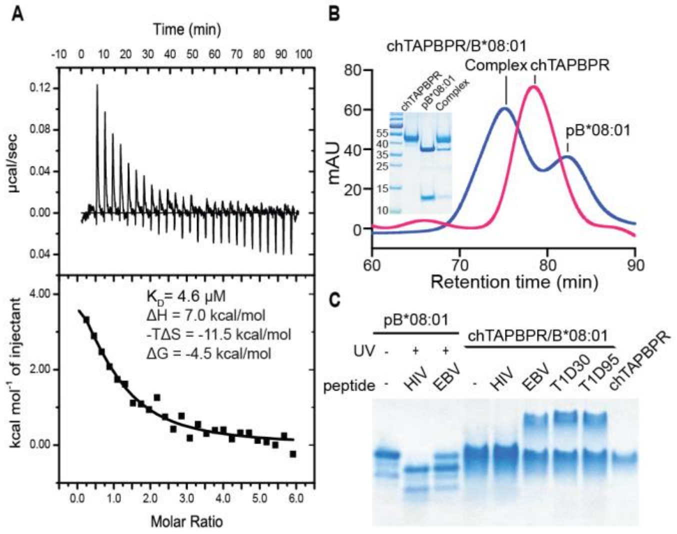

Figure S11. Chicken TAPBPR interacts with both peptide-loaded and peptide-receptive HLA-B*08:01 molecules. (A), Representative ITC titration of HLA-B*08:01/FLRGRAJGL (150 μM) with 3-fold excess peptide FLRGRAJGL (450 μM) into a sample containing 5 μM chTAPBPR with 450 μM FLRGRAJGL. The black line is the fit of the isotherm. Fitted values for K_D, ΔH, -TΔS, and ΔG were determined using a 1-site binding model. **(B),** SEC analysis of chTAPBPR (in pink) and the mixture of chTAPBPR and HLA-B*08:01/FLRGRAJGL after 40-minute UV irradiation (in blue). The peaks of chTAPBPR, HLA-B*08:01/FLRGRAJGL (pB*08:01), and chTAPBPR/HLA-B*08:01 complex were collected, concentrated, and ran on SDS/PAGE, which confirmed the identity of the complex peak. **(C),** The electrophoretic mobility shift assay (EMSA) of HLA-B*08:01/FLRGRAJGL (lane 1) and UV-irradiated HLA-B*08:01 with a 10-fold molar excess of a non-specific peptide AIFQSSMTK (HIV, lane 2) or a high-affinity peptide FLRGRAYGL (EBV, lane 3). Peptide-receptive chTAPBPR/HLA-B*08:01 complexes were loaded alone (lane 4) and with a 10-fold molar excess of the non-specific peptide HIV (lane 5) or the high-affinity peptides, EBV (lane 6), ALWMRLLPL (T1D30, lane 7), and MLYQHLLPL (T1D95, lane 8). ChTAPBPR alone was also loaded as a control (lane 10).

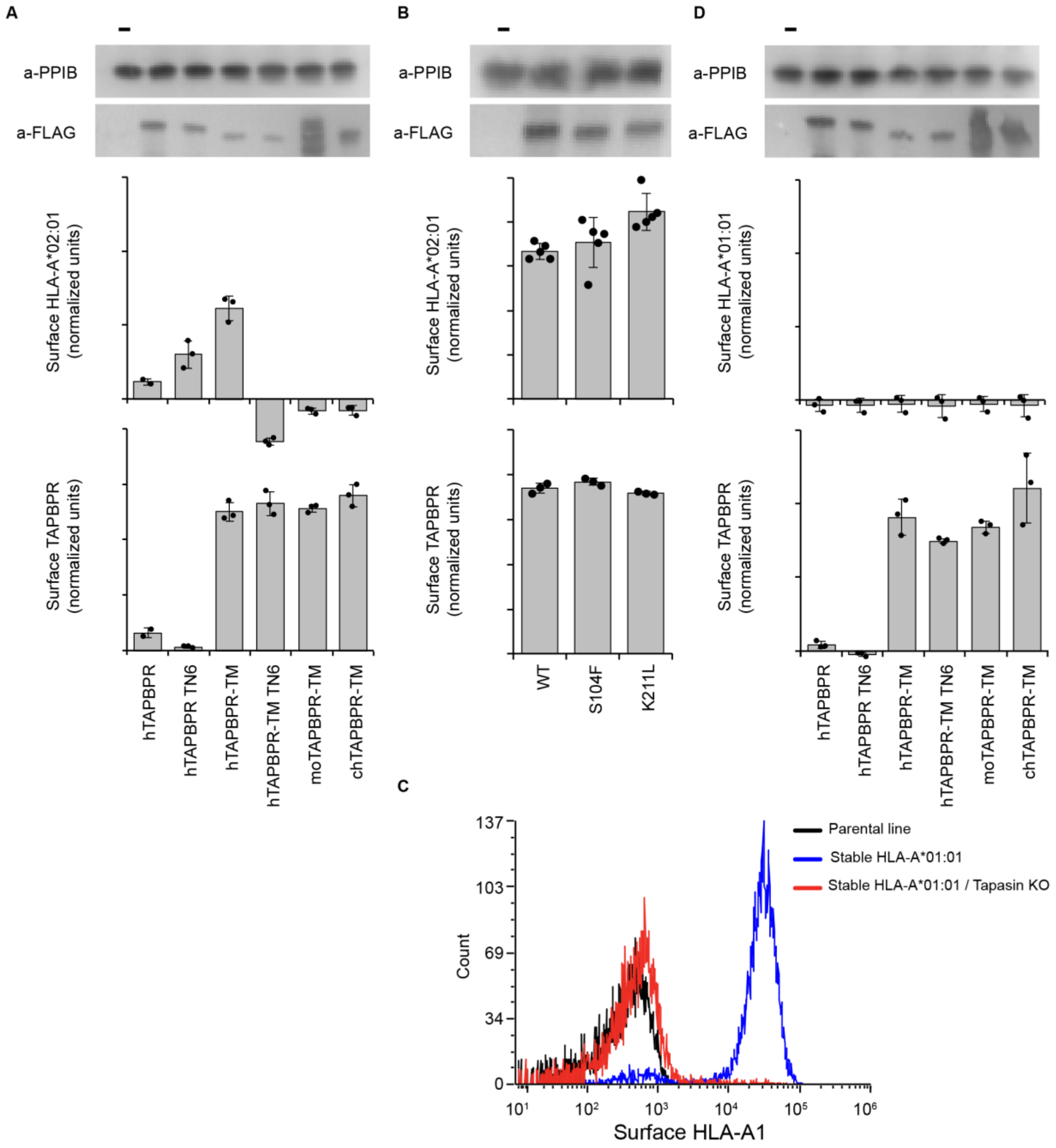

Figure S12. TAPBPR-TM promotes surface expression of HLA-A*02:01. (A), Tapasin-KO Expi293F cells were transiently transfected with FLAG-tagged TAPBPR. (Top) Western blots show total expression levels of TAPBPR proteins. The housekeeping enzyme PPIB was detected as a loading control. (Center) Surface levels of endogenous HLA-A*02:01 were measured by flow cytometry. Fluorescence levels of vector-only transfected cells were subtracted. hTAPBPR

mediates a small increase in surface HLA-A*02:01 that is not disrupted by the TN6 mutations, whereas hTAPBPR-TM mediates a large increase in surface HLA-A*02:01 that is decreased by the TN6 mutations. The data are consistent with intracellular TAPBPR proteins interacting with nascent HLA-A*02:01 (and sequestering a pool of HLA-A*02:01 intracellularly) in a manner independent of the TN6 mutations, whereas surface interactions are diminished by the TN6 mutations. (Bottom) Surface levels of FLAG-tagged TAPBPR measured by flow cytometry. TAPBPR with a native C-terminus is primarily intracellular, whereas TAPBPR-TM proteins traffic to the plasma membrane. Data are mean ± σ, n = 3 independent replicates. **(B),** Tapasin-KO Expi293F cells were transfected with WT, S104F, and K211L hTAPBPR-TM. (Top) Total expression of hTAPBPR-TM proteins by western blot, (center) surface expression of HLA-A*02:01 by flow cytometry, and (bottom) surface expression of hTAPBPR-TM proteins by flow cytometry. Data are mean ± σ, n = 5 independent replicates. **(C),** To generate an HLA-A*01:01 positive/tapasin-KO line, Expi293F cells (parental, black) were stably transfected with untagged HLA-A*01:01 (blue). The tapasin gene was then targeted by Cas9 as previously described to create the final line (red). Cells were stained with anti-HLA-A*01:01 for flow cytometry analysis. **(D),** HLA-A*01:01 positive/tapasin-KO Expi293F cells were transfected with TAPBPR constructs. (Top) Total expression of TAPBPR proteins by western blot, (center) surface expression of HLA-A*01:01 by flow cytometry, and (bottom) surface expression of TAPBPR proteins by flow cytometry. The TAPBPR proteins have no effect on surface HLA-A*01:01 levels. Data are mean ± σ, n = 3 independent replicates.

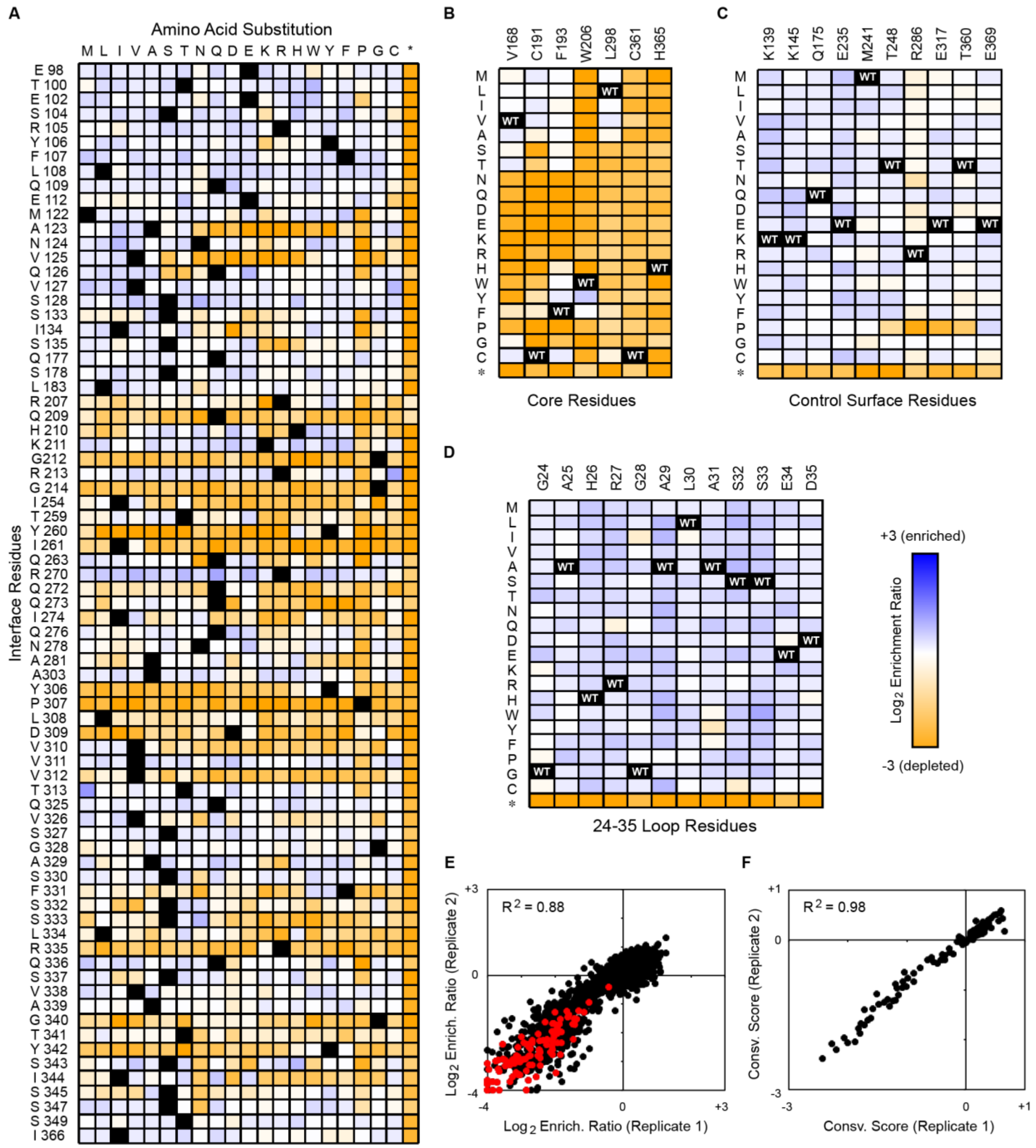

Figure S13. The mutational landscape of human TAPBPR-TM based on promoting surface expression of HLA-A*02:01. (A), Log_2 enrichment ratios from the deep mutational scan are plotted from ≤ -3 (orange, deleterious/depleted mutations) to ≥ +3 (blue, enriched mutations). Amino acid substitutions are on the horizontal axis, hTAPBPR-TM residue position is on the

vertical axis. *, stop codon. Wild type amino acids are in black. **(B)-(D),** Heat maps plot the $\log_2$ enrichment ratios from ≤ -3 (orange) to ≥ +3 (blue) for mutations in hTAPBPR-TM in the **(B)** core, **(C)** on the surface away from the MHC-I interface, and **(D)** in the 24-35 loop. Amino acid substitutions are on the vertical axes, hTAPBPR-TM residue positions are on the horizontal axes. **(E),** $\log_2$ enrichment ratios for each mutation in hTAPBPR-TM are plotted from two independent sorting experiments. R^2 is indicated for the agreement between replicates for missense mutations in black. Nonsense mutations are red. **(F),** Conservation scores, calculated by averaging the $\log_2$ enrichment ratios for all amino acid substitutions at a given hTAPBPR-TM residue, are plotted, showing close agreement between two independent sorting experiments.

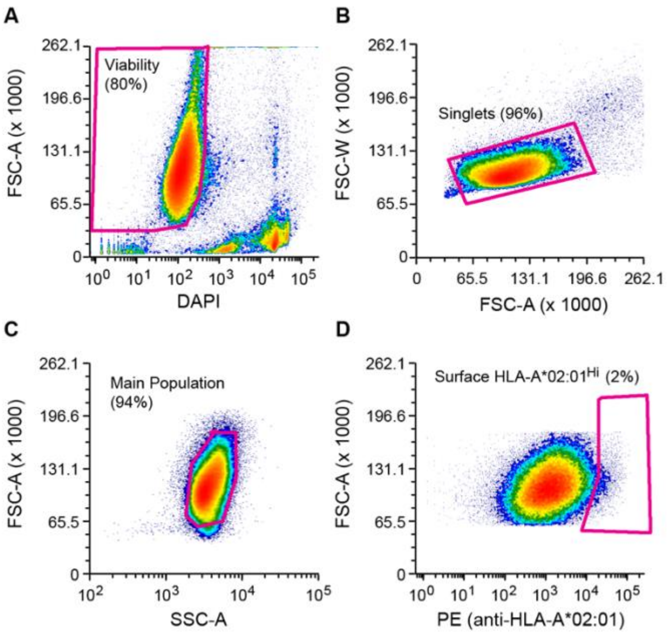

Figure S14. Sorting strategy for the deep mutational scan of TAPBPR-TM. Tapasin-KO Expi293F cells were transfected with a plasmid library encoding TAPBPR-TM mutants, under conditions previously shown to result in cells typically expressing no more than a single coding sequence. Flow cytometry histograms show how the transfected cell cultures were gated for FACS to enrich for cells expressing TAPBPR-TM mutants that promote surface HLA-A*02:01 localization. Cells were gated for viability **(A,** DAPI negative**)**, by forward scattering (FSC-A/area and FSC-W/width) to exclude doublets **(B)**, by forward (FSC) and side (SSC) scattering for the main population **(C)**, and by fluorescence for high levels of staining with anti-HLA-A*02:01 PE conjugate **(D)**. Gates are shown in magenta and the percentages of events that are gated are in parentheses.

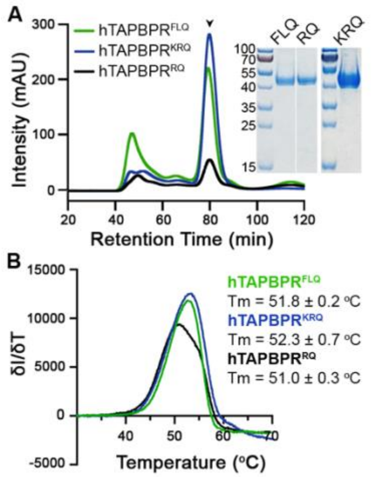

Figure S15. Purification of recombinant hTAPBPR mutants. (A), SEC traces of hTAPBPR S104F K211L R270Q (hTAPBPR^{FLQ}), R105K K211R R270Q (hTAPBPR^{KRQ}), and K211R R270Q (hTAPBPR^{RQ}) mutants. The protein peak is indicated by the arrow and is further confirmed by SDS/PAGE analysis. **(B),** DSF of hTAPBPR (T_m = 53.0 °C, red) relative to hTAPBPR^{FLQ} (T_m = 51.8 °C, green), hTAPBPR^{KRQ} (T_m = 52.3 °C, blue), and hTAPBPR^{RQ} (T_m = 51.0 °C, black).

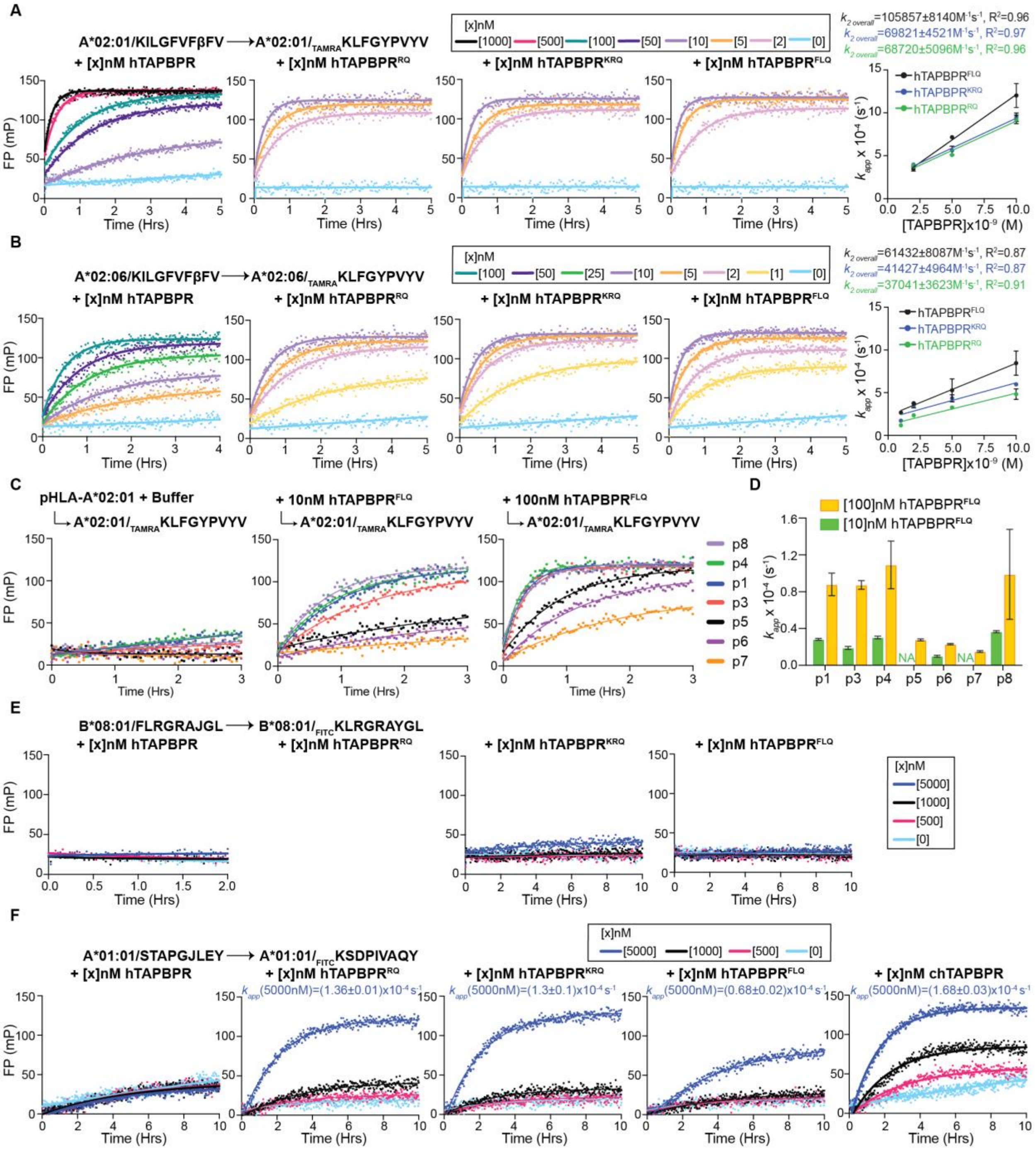

Figure S16. Human TAPBPR mutants show enhanced peptide exchange kinetics on HLA-A*02 and enable exchange on HLA-A*01:01 molecules. (A)-(B), The association profiles of fluorophore-conjugated peptide $_{\text{TAMRA}}$KLFGYPVYV to **(A)** HLA-A*02:01/KILGFVFβFV and **(B)** HLA-A*02:06/KILGFVFβFV in the presence of hTAPBPR, hTAPBPR^{RQ}, hTAPBPR^{KRQ}, and

hTAPBPR^{FLQ} at various concentrations, as indicated. Results of three replicates (mean) are plotted. The data were fitted to a monoexponential association model to determine apparent rate constants k_{app}. Linear correlations between the apparent rate constants k_{app} and the concentrations of TAPBPR orthologs were determined. The extrapolation of the slope determines the overall rate $k_{2overall}$. Results of three replicates (mean ± σ) are plotted. **(C),** The association profiles of $_{TAMRA}$KLFGYPVYV to HLA-A*02:01/βFILGFVFTV (p1), KIβFGFVFTV (p3), KILβFFVFTV (p4), KILGβFVFTV (p5), KILGFβFFTV (p6), KILGFVβFTV (p7), KILGFVFβFV (p8) without hTAPBPR^{FLQ} (buffer), with 10 nM or 100 nM hTAPBPR, as indicated. The data were fitted to a monoexponential association model to determine the apparent rate constant k_{app}. Results of three replicates are plotted. **(D),** The comparison of k_{app} for fluorescent peptide binding to HLA-A*02:01/KILGFVFTV with βF substitution at indicated positions in the presence of 10nM or 100nM hTAPBPR. The apparent rate constant k_{app} was determined by fitting the raw trace to a monoexponential association model. Results of three replicates (mean ± σ) are plotted. **(E),** The association profiles of fluorophore-conjugated peptide $_{FITC}$KLRGRAYGL to HLA-B*08:01/FLRGRAJGL in the presence of hTAPBPR, hTAPBPR^{KRQ}, and hTAPBPR^{FLQ} at various concentrations, as indicated. Results of three replicates (mean) are plotted. **(F),** The association profiles of fluorophore-conjugated peptide $_{FITC}$KSDPIVAQY to HLA-A*01:01/STAPGJLEY in the presence of hTAPBPR, hTAPBPR^{RQ}, hTAPBPR^{KRQ}, hTAPBPR^{FLQ}, and chTAPBPR at various concentrations, as indicated. The data were fitted to a monoexponential association model to determine apparent rate constants k_{app}. Results of three replicates (mean ± σ) are plotted.

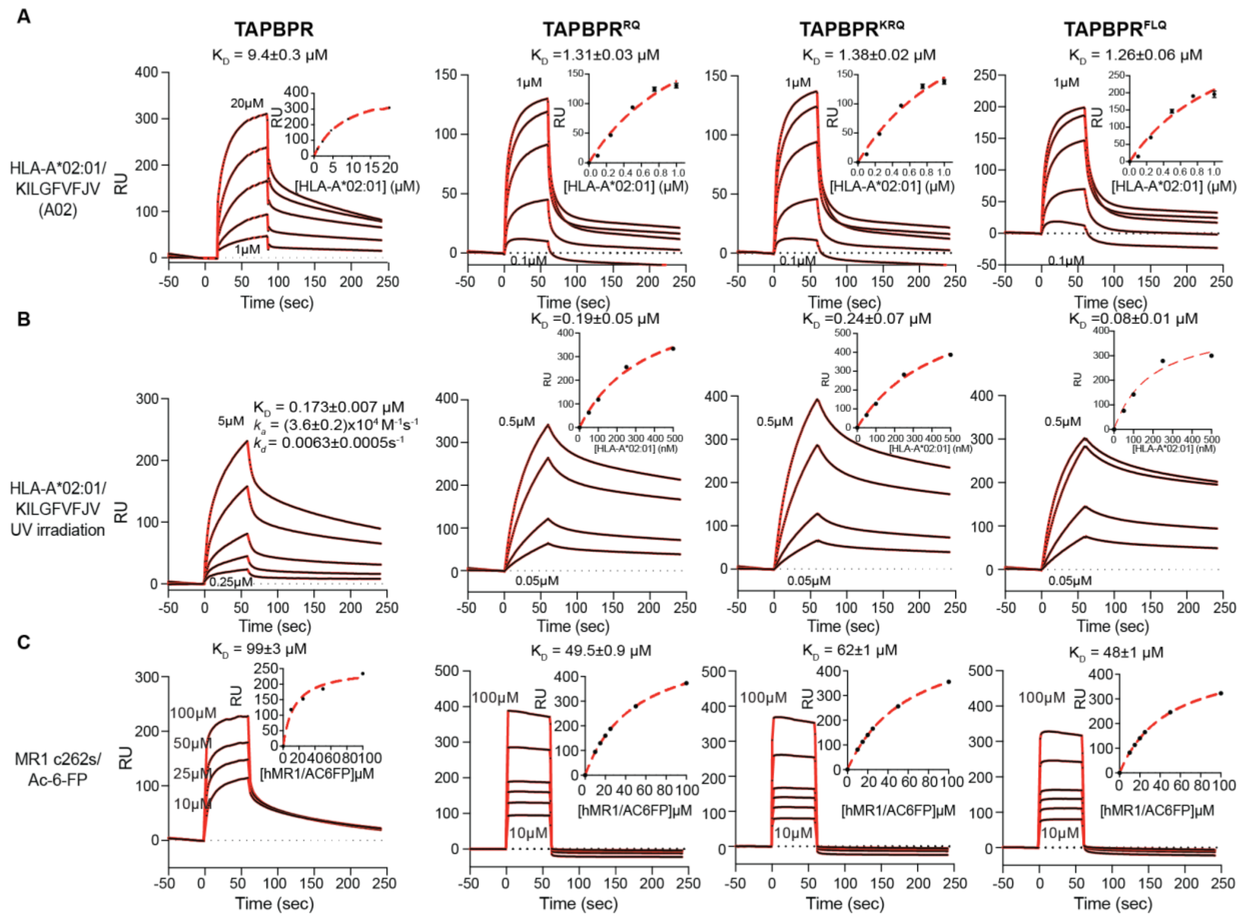

Figure S17. Assessment of TAPBPR mutations on the interactions with classical and nonclassical MHC-I molecules. (A), Representative SPR sensorgrams of various concentrations of peptide-loaded HLA-A*02:01 flowed over a streptavidin chip coupled with hTAPBPR, hTAPBPR^{RQ}, hTAPBPR^{KRQ}, or hTAPBPR^{FLQ}-biotin. **(B),** Representative SPR sensorgrams of various concentrations of peptide-deficient (empty) HLA-A*02:01 flowed over a streptavidin chip coupled with hTAPBPR, hTAPBPR^{RQ}, hTAPBPR^{KRQ}, or hTAPBPR^{FLQ}-biotin. **(C),** Representative SPR sensorgrams of various concentrations of Ac-6-FP loaded MR1 C262S flowed over a streptavidin chip coupled with hTAPBPR, hTAPBPR^{RQ}, hTAPBPR^{KRQ}, or hTAPBPR^{FLQ}-biotin. The concentrations of analyte for the top and the bottom sensorgrams are noted. Data are

mean ± σ for n = 2 technical replicates. K_D, equilibrium constant; k_a, association rate constant; k_d, dissociation rate constant; RU, resonance units.

Table S1. Mean fluorescence intensity (MFI) ratio of human and chicken TAPBPR relative to the control W6/32 experiments. Alleles with ratios above the threshold (MFI ratio=5.97) are in bold.

	**MHC-I MFI ratio**	
**Allotype**	hTAPBPR	chTAPBPR
A*01:01	3.90	2.67
**A*02:01**	**39.68**	3.88
**A*02:03**	**19.10**	3.28
**A*02:06**	**12.75**	2.97
A*03:01	4.38	5.63
A*11:01	3.63	3.11
A*11:02	4.63	4.09
**A*23:01**	**49.93**	**9.25**
**A*24:02**	**20.32**	**20.80**
**A*24:03**	**15.50**	**22.43**
A*25:01	5.20	4.77
A*26:01	1.79	2.32
**A*29:01**	3.88	**11.42**
**A*29:02**	**74.26**	**528.17**
A*30:01	3.75	3.08
A*30:02	2.88	1.80
A*31:01	3.69	2.73
**A*32:01**	**8.71**	**15.37**
A*33:01	2.63	1.56
A*33:03	2.00	2.05
A*34:01	3.87	3.52
A*34:02	4.00	3.50
A*36:01	3.50	2.50
A*43:01	2.39	2.23
A*66:01	1.66	2.30
A*66:02	2.32	3.19
**A*68:01**	**6.13**	3.04
**A*68:02**	**115.17**	4.45
**A*69:01**	**10.17**	2.33
**A*74:01**	**6.08**	**7.53**
A*80:01	2.88	2.10
B*07:02	2.58	2.41

	**MHC-I MFI ratio**	
**Allotype**	hTAPBPR	chTAPBPR
**B*08:01**	3.38	**16.59**
B*13:01	1.79	1.70
B*13:02	2.50	1.56
B*14:01	2.93	2.15
B*14:02	2.43	1.58
B*15:01	3.28	1.39
B*15:02	2.48	1.87
B*15:03	2.31	1.46
B*15:10	2.59	4.20
B*15:11	1.55	1.39
B*15:13	2.34	3.54
B*15:16	2.71	3.18
B*18:01	1.95	1.24
B*27:05	3.23	1.77
B*27:08	2.29	1.21
B*35:01	1.97	1.55
**B*37:01**	**50.02**	**1126.35**
B*38:01	3.13	5.91
B*39:01	3.35	1.88
B*40:01	1.62	1.27
B*40:02	2.44	1.39
B*40:06	1.82	1.31
B*41:01	2.39	2.14
B*42:01	2.79	2.09
B*44:02	2.38	2.63
B*44:03	2.14	2.16
B*45:01	2.86	2.23
B*46:01	2.21	1.80
B*47:01	3.00	2.60
B*48:01	1.88	2.16
B*49:01	3.24	2.04
B*50:01	2.43	1.62

	**MHC-I MFI ratio**	
**Allotype**	hTAPBPR	chTAPBPR
B*51:01	3.10	3.32
B*51:02	3.14	3.95
B*52:01	2.48	2.43
B*53:01	1.83	2.12
B*54:01	2.07	1.80
B*55:01	1.74	1.47
B*56:01	2.81	1.67
B*57:01	4.33	2.86
**B*57:03**	2.74	**6.79**
B*58:01	2.09	1.95
**B*59:01**	2.71	**6.49**
B*67:01	3.06	1.87
B*73:01	4.86	5.09
B*78:01	2.28	1.64
B*81:01	1.73	1.82
B*82:01	1.63	1.38
**C*01:02**	2.49	**6.40**
C*02:02	1.84	1.49
C*03:02	2.64	1.95
C*03:03	2.56	5.46
**C*03:04**	2.65	**8.72**
C*04:01	1.65	1.76
C*05:01	1.85	1.34
C*06:02	1.36	1.59
C*07:02	1.58	1.32
C*08:01	1.62	1.12
C*12:03	1.57	2.00
C*14:02	1.71	1.36
C*15:02	2.00	1.52
C*16:01	1.60	1.29
C*17:01	1.83	1.59
C*18:02	1.58	1.64

Table S2. Summary of peptides used in this study.

Peptide Name	HLA Allotype	Sequence
PhotoA01	A*01:01	STAPGJLEY
PhotoA02	A*02:01	KILGFVFJV
PhotoA24	A*24:02	VYGJVRACL
PhotoA30	A*30:01	AIFQSSMJK
PhotoA29	A*29:02	VFAQVKQJY
PhotoA68	A*68:02	SVYDFFVJL
PhotoA11	A*11:01	KLIETYFJK
PhotoB08	B*08:01	FLRGRAJGL
PhotoB37	B*37:01	FEDLRVJSF
PhotoE01	E*01:03	VMAPJTLVL
PhotoG01	G*01:01	VMAPJTLVL
BetaA02	A*02:01	KILGFVF(βF)V
BetaA03_A11	A*03:01 & A*11:01	KLIETYF(βF)K
BetaA30	A*30:01	KTFPPTE(βF)K
BetaA68	A*68:02	ETAGIGIL(βF)V
BetaB37	B*37:01	FEDLRV(βF)SF
FITCA01	A*01:01	FITC-KSDPIVAQY
FITCA30	A*30:01	FITC-KTFPPTEPK
TAMRAA02	A*02:01 & A*68:02	TAMRA-KLFGYPVYV
TAMRAA24	A*24:02	TAMRA-KYNPIRTTF
FITCA03_A11	A*03:01 & A*11:01	FITC-KLIETYFSK
FITCA29	A*29:02	FITC-KLIDVFHQY
FITCB08	B*08:01	FITC-KLRGRAYGL
FITCB37	B*37:01	FITC-KEDLRVSSF
Phox2b	A*24:02	QYNPIRTTF
TAX9	A*02:01	LLFGYPVYV
p29	A*02:01 & A*01:01 nonbinder	YPNVNIHNF
RAS	A*01:01	ILDTAGQEEY
Titin	A*01:01	ESDPIVAQY
SARS P37	A*01:01	CTDDNALAYY
SARS P39	A*01:01	PTDNYITTY
SARS P44	A*29:02	FTSDYYQLY
HCMV	B*08:01	RPHERNGFTVL
EBV	B*08:01	FLRGRAYGL
T1D30	B*08:01	ALWMRLLPL
T1D95	B*08:01	MLYQHLLPL
HIV	B*08:01 nonbinder	AIFQSSMTK
NP338	B*37:01	FEDLRVLSF
CMV pp65	B*37:01 nonbinder	NLVPMVATV

Table S3. Summary of HLA-A*02:01 loaded with KILGFVFTV-derived peptides comprising substitutions of different residues with βF and micropolymorphic HLA-A*02 subtypes loaded with KILGFVFβFV.

**HLA-A*02 subtypes**	**T_m (ºC)**
A*02:01/βFILGFVFTV	53.9
A*02:01/KIβFGFVFTV	58.1
A*02:01/KILβFFVFTV	54.3
A*02:01/KILGβFVFTV	61.8
A*02:01/KILGFβFFTV	60.7
A*02:01/KILGFVβFTV	63.3
A*02:01/KILGFVFβFV	53.2
A*02:01/BetaA02	52.6
A*02:06/BetaA02	53.1
A*02:11/BetaA02	53.8
A*02:71/BetaA02	54.2
A*02:77/BetaA02	54.2

Table S4. Summary of MHC-I residues contacting TAPBPR for selected HLA alleles used in this study, including HLA-A*01:01, A*30:01, A*29:02, A*02:01, A*68:02, A*03:01, A*11:01, A*24:02, B*08:01, B*37:01, E*01:03, and G*01:01.

**HLA Allele**	**Amino Acid Position** [84, 86, 113, 115, 120, 122, 127, 128, 134, 135, 136, 138, 141, 142, 144, 145, 148, 225, 228, 229, 230, 231, 232, 244]
A*01:01	YNYQGDNETAAMQIKRETTELVEW
A*30:01	YNYQGDNETAAMQIQRETTELVEW
A*29:02	YNYQGDNETAAMQIQRETTELVEW
A*02:01	YNYQGDKETAAMQTKHETTELVEW
A*68:02	YNYQGDKETAAMQTKHETTELVEW
A*03:01	YNYQGDNETAAMQIKRETTELVEW
A*11:01	YNYQGDNETAAMQIKRETTELVEW
A*24:02	YNYQGDKETAAMQIKRETTELVEW
B*08:01	YNHQGDNETAATQIQRETTELVEW
B*37:01	YNYQGDNETAATQIQRETTELVEW
E*01:03	YNYQGDNETAVTQIEQNTTELVEW
G*01:01	YNYQGDNETAATQIKRETVELVEW
